# LncRNA MIR17HG promotes colorectal cancer liver metastasis by mediating a glycolysis-associated positive feedback circuit

**DOI:** 10.1038/s41388-021-01859-6

**Published:** 2021-06-18

**Authors:** Senlin Zhao, Bingjie Guan, Yushuai Mi, Debing Shi, Ping Wei, Yanzi Gu, Sanjun Cai, Ye Xu, Xinxiang Li, Dongwang Yan, Mingzhu Huang, Dawei Li

**Affiliations:** 1 Department of Colorectal Surgery, Fudan University Shanghai Cancer Center, Shanghai, China; 2 Department of Oncology, Shanghai Medical College, Fudan University, Shanghai, China; 3 Department of General Surgery, Shanghai General Hospital, School of Medicine, Shanghai Jiaotong University, Shanghai, China; 4 Department of Gastrointestinal Surgery, The Second Hospital, Cheeloo College of Medicine, Shandong University, Jinan, China; 5 Cancer Institute, Fudan University Shanghai Cancer Center, Shanghai, China; 6 Department of Pathology, Fudan University Shanghai Cancer Center, Shanghai, China; 7 Department of Biobank, Fudan University Shanghai Cancer Center, Shanghai, China; 8 Department of Medical Oncology, Fudan University Shanghai Cancer Center, Shanghai, China

**Keywords:** Cancer metabolism, Colorectal cancer, Cell migration

## Abstract

Glycolysis plays a crucial role in reprogramming the metastatic tumor microenvironment. A series of lncRNAs have been identified to function as oncogenic molecules by regulating glycolysis. However, the roles of glycolysis-related lncRNAs in regulating colorectal cancer liver metastasis (CRLM) remain poorly understood. In the present study, the expression of the glycolysis-related lncRNA MIR17HG gradually increased from adjacent normal to CRC to the paired liver metastatic tissues, and high MIR17HG expression predicted poor survival, especially in patients with liver metastasis. Functionally, MIR17HG promoted glycolysis in CRC cells and enhanced their invasion and liver metastasis in vitro and in vivo. Mechanistically, MIR17HG functioned as a ceRNA to regulate HK1 expression by sponging miR-138-5p, resulting in glycolysis in CRC cells and leading to their invasion and liver metastasis. More interestingly, lactate accumulated via glycolysis activated the p38/Elk-1 signaling pathway to promote the transcriptional expression of MIR17HG in CRC cells, forming a positive feedback loop, which eventually resulted in persistent glycolysis and the invasion and liver metastasis of CRC cells. In conclusion, the present study indicates that the lactate-responsive lncRNA MIR17HG, acting as a ceRNA, promotes CRLM through a glycolysis-mediated positive feedback circuit and might be a novel biomarker and therapeutic target for CRLM.

## Introduction

Colorectal cancer (CRC) accounts for ~10% of all cancer cases and deaths worldwide [1]. Despite the great progress made in the treatment of CRC, ~25% of patients present with liver metastatic disease at the time of initial diagnosis, and another 25% of patients diagnosed with early CRC eventually progress to liver metastasis [2, 3]. The 5-year median overall survival time of these individuals is only ~30 months, indicating that liver metastasis is one of the main causes of poor prognosis in CRC patients for lacking of efficient biomarkers and knowledge about their roles in liver metastasis [4, 5]. Therefore, discovering key drivers and revealing the underlying mechanisms of liver metastasis are still urgent.

CRCs and other solid tumors can undergo alterations in their major metabolic phenotypes from mitochondrial oxidation to glycolysis even in the presence of abundant oxygen, a process termed aerobic glycolysis or the Warburg effect [6]. This rewired glucose metabolism is a hallmark of tumor biology required for carcinogenesis, tumor progression, and drug resistance [7–10]. Aerobic glycolysis is universally involved in rapid ATP synthesis, tumor microenvironment alterations, biosynthesis promotion, and cell signaling activation [11, 12]. Although the essential role of aerobic glycolysis in cancers has been widely investigated, roles of glycolysis in colorectal cancer liver metastasis (CRLM) remain poorly understood. Accordingly, clarification of this mechanism is necessary for understanding the process of liver metastasis in CRC.

Long noncoding RNAs (lncRNAs) are a class of RNAs broadly defined as transcripts that are longer than 200 nucleotides and evolutionarily conserved [13, 14]. Previously considered transcriptional noise, these mRNA-like transcripts frequently contain a 5′ cap and 3′ poly(A) tail but do not have protein-coding ability, a characteristic partially attributed to their lack of translated open reading frames [15]. In malignancy development, lncRNAs have been considered diagnostic and prognostic biomarkers in solid tumors [16–19]. We previously reported that the lncRNA MALAT1 could sponge miR-106b-5p to promote the progression of CRC [20]. In addition, we used weighted gene coexpression network analysis (WGCNA) to identify differentially expressed lncRNAs and mRNAs in the adjacent normal tissues, CRC tissues, and paired liver metastatic tissues of eight patients who underwent simultaneous resection of the CRC tumor and liver metastasis and did not receive any preoperative treatment. Then, we constructed a lncRNA–mRNA network for the pathway involved in aerobic glycolysis. We found that the expression of the glycolysis-related lncRNA MIR17HG increased gradually from adjacent normal tissues to CRC tissues to their paired liver metastatic tissues in CRC patients. Whether lncRNA MIR17HG can promote CRLM by regulating glycolysis needs further exploration.

In this study, we revealed that (1) high MIR17HG expression predicted poor prognosis in CRC patients, especially in patients with liver metastasis; (2) MIR17HG functioned as a ceRNA to regulate HK1 expression by sponging miR-138-5p, which promoted aerobic glycolysis and liver metastasis in CRC; and (3) glycolysis-accelerated lactate accumulation promoted MIR17HG transcription via the p38/Elk-1 pathway, thus forming a positive feedback loop. These findings demonstrated the roles of lncRNA MIR17HG in CRLM and highlighted its significance for predicting the risk of CRLM.

## Results

### Comprehensive WGCNA identified the glycolysis-related lncRNA MIR17HG as an oncogene involved in colorectal cancer liver metastasis

**Fig. 1:**
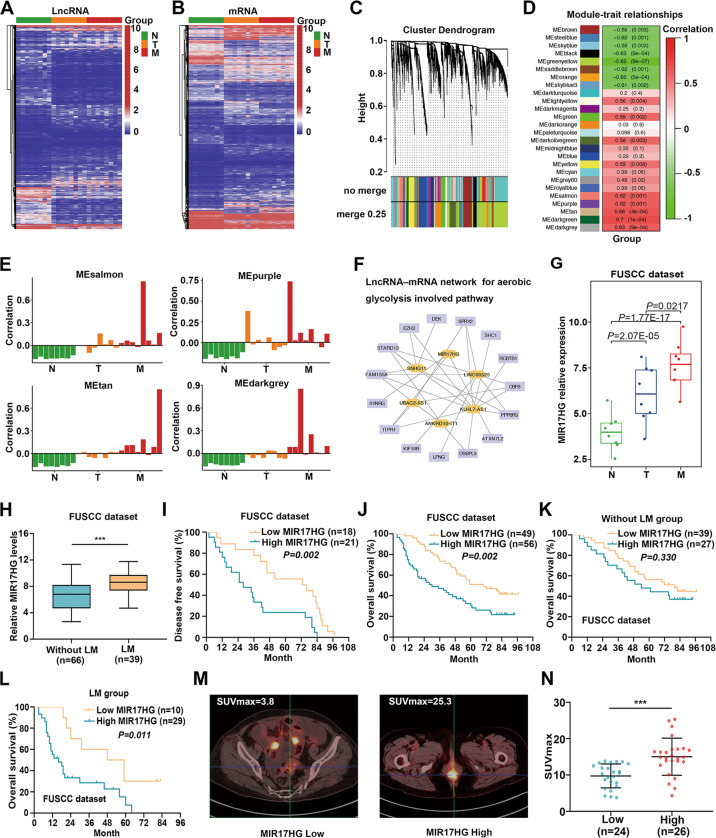
Comprehensive WGCNA identified the glycolysis-related lncRNA MIR17HG as an oncogene during colorectal cancer liver metastasis. **A**, **B** A total of eight pairs of adjacent normal, CRC and liver metastatic tissues were subjected to lncRNA (**A**) and mRNA (**B**) microarray analysis. Heat maps showed differentially expressed genes (eight biological replicates); **C** Cluster dendrogram of modules identified by WGCNA of lncRNA and mRNA microarray data. **D** Associations of modules with CRLM; **E** Four modules (MEdarkgrey, MEpurple, MEsalmon and MEtan) were found to be significantly and gradually increased from adjacent normal to CRC to paired liver metastatic tissues (Student’s *t* test); **F** Comprehensive analysis of KEGG pathways involved in glycolysis in MEpurple; **G** lncRNA MIR17HG expression gradually increased from adjacent normal to CRC to paired liver metastatic tissues (*n* = 8 for each condition); **H** The expression levels of MIR17HG in colorectal cancer tissues from patients with and without liver metastasis in the FUSCC dataset; **I**, **J** Kaplan–Meier analysis with log-rank test for evaluating MIR17HG expression for predicting the disease-free survival (DFS, **I**) and overall survival (OS, **J**) of CRC patients in the FUSCC dataset; **K**, **L** Roles of MIR17HG expression in predicting the overall survival of patients without liver metastasis (**K**) and patients with liver metastasis (**L**); **M** Representative 18F-FDG PET/CT images of colorectal cancer patients with low or high MIR17HG expression; **N** Difference analysis of SUVmax in the MIR17HG-low and MIR17HG-high groups (***P < 0.001).

To identify key drivers involved in CRLM, microarray analysis was used to determine the lncRNA and mRNA expression profiles of adjacent normal, CRC, and paired liver metastatic tissues (*n* = 8 for each condition, Fig. 1A, B; Supplementary Table S1–6). Furthermore, WGCNA was applied to identify genes that played significant roles in CRLM. A total of 26 modules were identified, and 18 modules were significantly associated with CRLM (Fig. 1C, D, Supplementary Table S7). Student’s *t* test results revealed that the MEdarkgrey, MEpurple, MEsalmon, and MEtan modules were gradually increased from the adjacent normal tissues to the CRC tissues to the paired liver metastatic tissues from the same patient (Fig. 1E). Although another four modules showed decreases from adjacent normal to CRC to paired liver metastatic tissues, no significance was found between the groups of CRC and paired liver metastatic tissues (Supplementary Fig. S1a). Then, we used the gradually increased MEdarkgreen, MEpurple, MEsalmon, and MEtan modules for our subsequent study. Kyoto Encyclopedia of Genes and Genomes (KEGG) analyses based on the differentially expressed lncRNAs and mRNAs involved in the above four modules with gradual increases were used to identify the glycolysis-related pathways. Only the differentially expressed genes in the MEpurple module showed enrichment approaching significance in glycolysis-associated pathways (*P* = 0.066). Then, the differentially expressed lncRNAs and mRNAs in the MEpurple module were subjected to comprehensive network analyses of involvement in glycolysis-related pathways, and we found that 6 lncRNAs were associated with 15 mRNAs involved during CRLM in glycolysis pathways enriched in the MEpurple module (Fig. 1F).

More interestingly, the expression patterns of the six lncRNAs involved in glycolysis pathways showed that the expression of only lncRNA MIR17HG gradually increased from adjacent normal to CRC to paired liver metastatic tissues (Fig. 1G, Supplementary Fig. S1b-f). Furthermore, our data showed that MIR17HG expression was significantly higher in tumor tissues associated with liver metastasis than in liver metastasis-free tissues (Fig. 1H). Kaplan–Meier analysis with the log-rank test showed that high MIR17HG expression implied poor disease-free survival (DFS) and overall survival (OS) in CRC patients (Fig. 1I, J). Notably, although patients with high MIR17HG expression showed a trend toward a shorter OS time in both the liver metastasis and metastasis-free groups, high MIR17HG expression indicated a significant reduction in OS only in the liver metastasis group (Fig. 1K, L). In addition, analyses of The Cancer Genome Atlas (TCGA) dataset showed that MIR17HG was significantly upregulated in CRC tissues and that high MIR17HG expression indicated poor recurrence-free survival in CRC patients (Supplementary Fig. S1g-h). Furthermore, correlation analysis showed obvious relationships between the MIR17HG level and distant metastasis as well as advanced pathologic stage (Supplementary Table S8). Accordingly, MIR17HG might play an essential role in CRC progression, especially during CRLM. Cancer cells often facilitate glucose uptake and glycolysis to support tumor progression, and the fluoro-2-D-deoxyglucose F18 (18F-FDG) PET/CT results in 50 CRLM patients were analyzed. We found that the SUVmax values in the MIR17HG-high group (15.0 ± 5.1, *n* = 26) were significantly higher than those in the MIR17HG-low group (4.9 ± 1.3, *n* = 24), suggesting enhanced glucose metabolism in tumor tissues with MIR17HG upregulation (Fig. 1M, N). Taken together, these results indicate that glycolysis-related lncRNA MIR17HG plays important roles in CRLM.

### MIR17HG enhances CRC cell glycolysis in an HK1-dependent manner

**Fig. 2:**
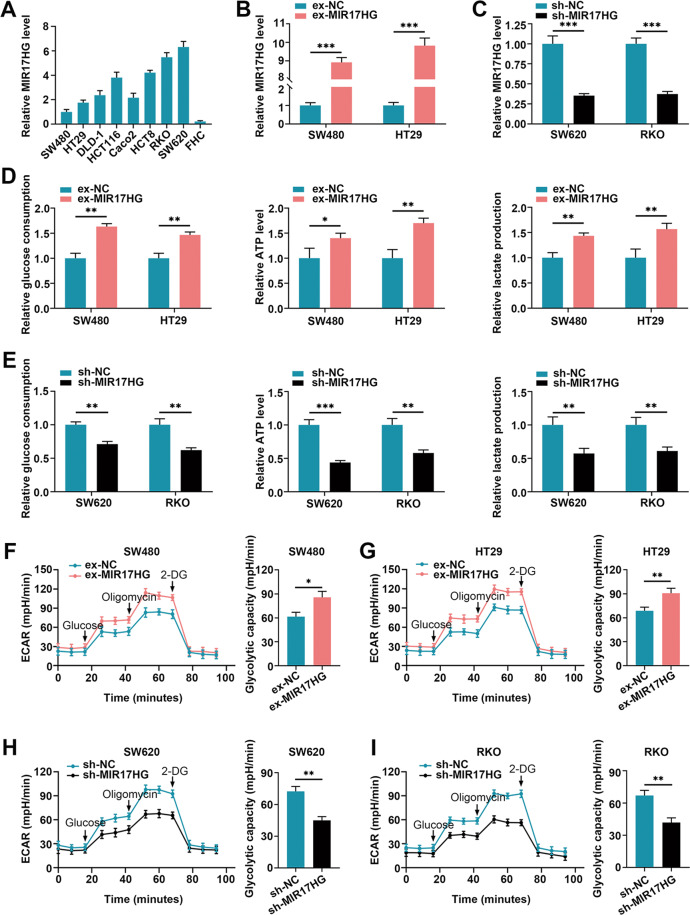
MIR17HG promotes glycolysis in colorectal cancer cells. **A** Expression levels of MIR17HG in eight colorectal cancer cell lines (Caco2, HT29, SW480, SW620, RKO, HCT116, HCT8, and DLD-1) and one normal colon cell line (FHC) were determined by quantitative RT-PCR. **B**–**C** The efficiency of MIR17HG overexpression (**B**) and knockdown (**C**) in the indicated cells was validated by quantitative RT-PCR. Controls (ex-NC or sh-NC). **D** Changes in relative glucose consumption, ATP levels and lactate production in SW480 and HT29 cells after MIR17HG overexpression; **E** Changes in relative glucose consumption, ATP levels and lactate production in SW620 and RKO cells upon MIR17HG knockdown; **F**, **G** Changes in ECAR levels in MIR17HG-overexpressing SW480 (**F**) and HT29 (**G**) cells. The ECAR after oligomycin treatment indicates glycolytic capacity; **H**, **I** Changes in glycolytic capacity upon MIR17HG knockdown in SW620 (**H**) and RKO (**I**) cells. *P < 0.05, **P < 0.01, ***P < 0.001.

Then, we investigated the role of MIR17HG in aerobic glycolysis of CRC cells. First, we evaluated the expression of MIR17HG in eight CRC cell lines and one normal colon cell line (FHC). The results indicated that the level of MIR17HG was higher in all CRC cell lines than in FHC cell line (Fig. 2A). Among these eight CRC cell lines, SW480/HT29 exhibited the lowest expression levels of MIR17HG, while SW620/ RKO showed the highest levels; thus, these cell lines were used to generate cells with MIR17HG overexpression or knockdown for further study (Fig. 2B, C). Then, we found that overexpression or silence of MIR17HG increased or decreased glucose consumption and ATP and lactate production in SW480/HT29 or SW620/ RKO cells, respectively (Fig. 2D, E). To further define the effect of MIR17HG on aerobic glycolysis in CRC cells, measurements of the extracellular acidification rate (ECAR) were performed. Consistent with the above results, MIR17HG overexpressing or downregulated cells exhibited an enhanced or weakened glycolysis phenotype with obvious increases or decreases in glycolytic capacity, respectively (Fig. 2F-I). Taken together, these results indicate that upregulation of MIR17HG reinforces aerobic glycolysis in CRC cells.

**Fig. 3:**
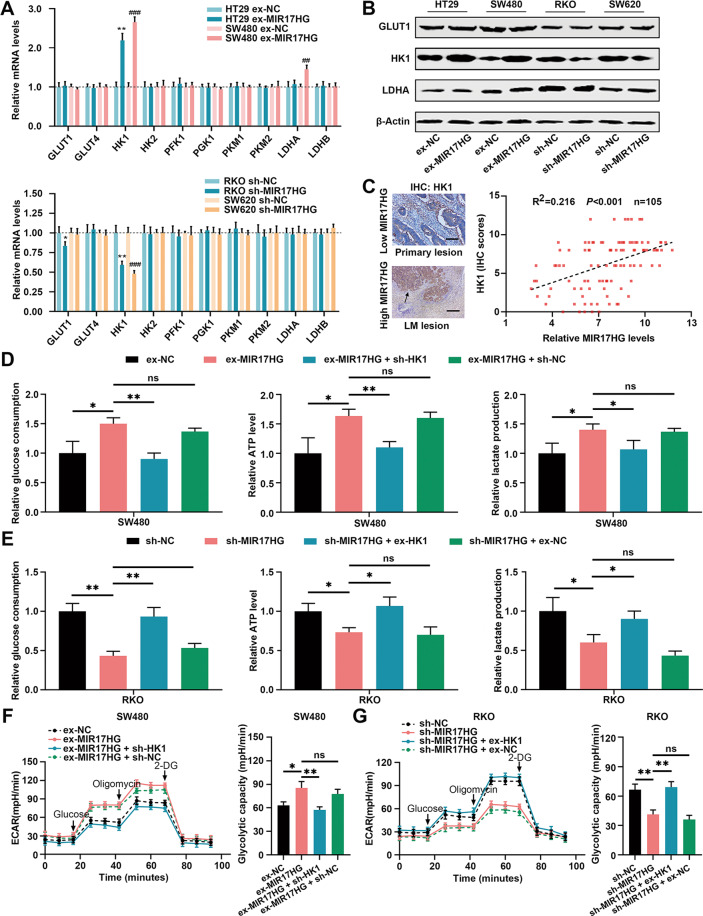
MIR17HG upregulates HK1 expression to promote glycolysis in colorectal cancer cells. **A** mRNA levels of a series of glycolysis-associated genes in MIR17HG-overexpressing HT29 and SW480 cells (upper panel) and in MIR17HG knockdown RKO and SW620 cells (lower panel); **B** Western blot analysis of GLUT1, HK1, and LDHA protein levels in MIR17HG-overexpressing HT29 and SW480 cells as well as in MIR17HG-knockdown RKO and SW620 cells, in which the mRNA levels differed significantly; **C** Representative HK1 immunohistochemical (IHC) images in CRC tissue with low MIR17HG expression and in liver metastasis tissue with high MIR17HG expression (left panel); Pearson correlation between MIR17HG and HK1 expression in 105 primary CRC tissues in the FUSCC dataset (right panel); original magnification, ×200; scale bar, 100 μm; **D** **E** Relative changes in glucose consumption, ATP levels and lactate production in MIR17HG-overexpressing SW480 cells (**D**) or MIR17HG-knockdown RKO cells (**E**) with or without HK1 overexpression; **F**, **G** ECAR values in MIR17HG-overexpressing SW480 cells with or without HK1 knockdown (**F**) and MIR17HG-knockdown RKO cells with or without HK1 overexpression (**G**). *P < 0.05; **P < 0.01; ***P < 0.001; ns not significant.

Glucose transporters (GLUT1/4) and glycolytic enzymes, including hexokinase (HK1/2), phosphofructokinase 1, phosphoglycerate kinase 1, pyruvate kinase (PKM1/2) and lactate dehydrogenase (LDHA/LDHB), are critical regulators of the glycolytic pathway [12]. We next investigated the expression profiles of these genes in MIR17HG overexpressing or downregulated cells. Interestingly, overexpression or knockdown of MIR17HG increased or decreased the HK1, LDHA and GLUT1 mRNA levels, respectively (Fig. 3A). Western blot analysis further confirmed that HK1 expression was significantly increased or decreased upon MIR17HG overexpression or silencing, respectively; however, no significant changes were found for GLUT1 and LDHA protein expression (Fig. 3B). In addition, Spearman correlation analysis showed that the HK1 immunohistochemical (IHC) staining score was positively correlated with the MIR17HG expression level in 105 CRC tissues, especially in liver metastatic tissues (Fig. 3C). These findings indicated that MIR17HG might regulate HK1 expression in CRC. Then, loss- and gain-of-function assays were performed by transfecting HK1 knockdown plasmids or overexpression vectors into MIR17HG-overexpressing or silenced cells, respectively. Unsurprisingly, knockdown or overexpression of HK1 in MIR17HG-overexpressing or silenced cells partially reversed the increases or decreases in ATP production, glucose uptake, and lactate production (Fig. 3D, E, Supplementary Fig. S2a-f). Moreover, ECAR measurements suggested that knockdown or overexpression of HK1 obviously offset the promotive or inhibiting effect of MIR17HG overexpression or knockdown on glycolytic capacity (Fig. 3F, G, Supplementary Fig. S2g-h). Therefore, MIR17HG promotes glycolysis in CRC cells in an HK1-dependent manner.

### MIR17HG promotes the invasion and liver metastasis of CRC cells in vitro and in vivo by upregulating HK1

**Fig. 4:**
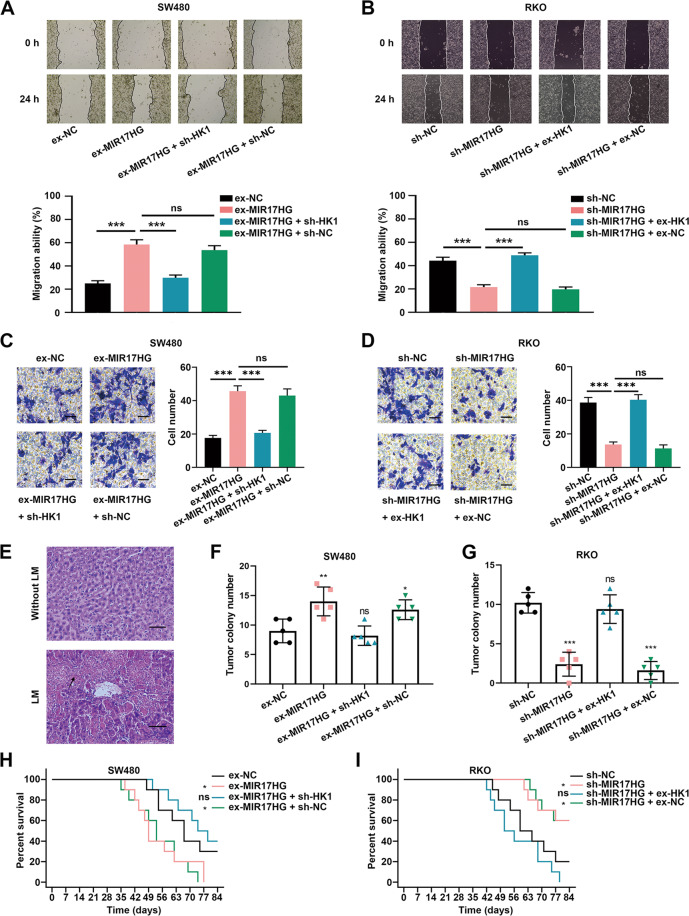
MIR17HG promotes the invasion and liver metastasis of CRC cells in vitro and in vivo by upregulating HK1 expression. **A**, **B** Effects of knockdown or overexpression of HK1 on the migration ability of MIR17HG-overexpressing SW480 cells (**A**) and MIR17HG-knockout RKO cells (**B**) were detected by a wound healing assay; **C**, **D** Effects of HK1 knockdown or overexpression on the invasive ability of MIR17HG-overexpressing SW480 cells (**C**) and MIR17HG-knockdown RKO cells (**D**) were determined by a transwell assay; **E** Representative HE images of liver tissues obtained from nude mice with (lower panel) or without (upper panel) liver metastasis, original magnification, ×200; scale bar, 50 μm; **F**, **G** The number of liver metastatic foci in five randomly selected mice of each group was counted under a microscope; **H**, **I** Overall survival of each group of mice injected with engineered SW480 (**H**) or RKO (**I**) cells (*n* = 10 for each group). *P < 0.05; **P < 0.01; ***P < 0.001; ns not significant.

Subsequently, we explored whether MIR17HG could promote the malignant phenotype of CRC cells in an HK1-dependent manner. Wound healing and transwell assays were performed to investigate effects of MIR17HG on the migration and invasion of CRC cells in vitro. The enhanced or suppressed wound healing upon overexpression or knockdown of MIR17HG was markedly weakened by silencing HK1 or enhanced by overexpressing HK1, respectively (Fig. 4A, B, Supplementary Fig. S3a-b). Moreover, the increase or decrease in the number of invaded CRC cells after overexpression or knockdown of MIR17HG was markedly abrogated by silencing or upregulating HK1 expression, respectively (Fig. 4C, D, Supplementary Fig. S3c-d). Accordingly, MIR17HG enhanced the migratory and invasive abilities of CRC cells in vitro in an HK1-dependent manner.

To explore the potential role of MIR17HG in CRLM in vivo, we transplanted engineered SW480 and RKO cells into nude mice via splenic injection and used hematoxylin-eosin (HE) staining to count metastatic foci in mouse livers (Fig. 4E). Similarly, overexpression or downregulation of MIR17HG could increase or decrease the number of liver metastatic lesions in mice and HK1 protein levels while these effects were significantly weakened upon knockdown or overexpression of HK1 (Fig. 4F, G, Supplementary Fig. S3e-f). More importantly, the OS time of mice was greatly reduced or increased in the MIR17HG overexpression group or knockdown group, as compared with controls, and these effects were partially dampened upon HK1 knockdown or overexpression, respectively (Fig. 4H, I). In addition, we found that HK1 expression was positively correlated with distant metastasis in clinic (Supplementary Table S9). Accordingly, these findings revealed that MIR17HG also promoted CRLM in an HK1-dependent manner in vivo.

### MIR17HG upregulates HK1 expression by sponging miR-138-5p

**Fig. 5:**
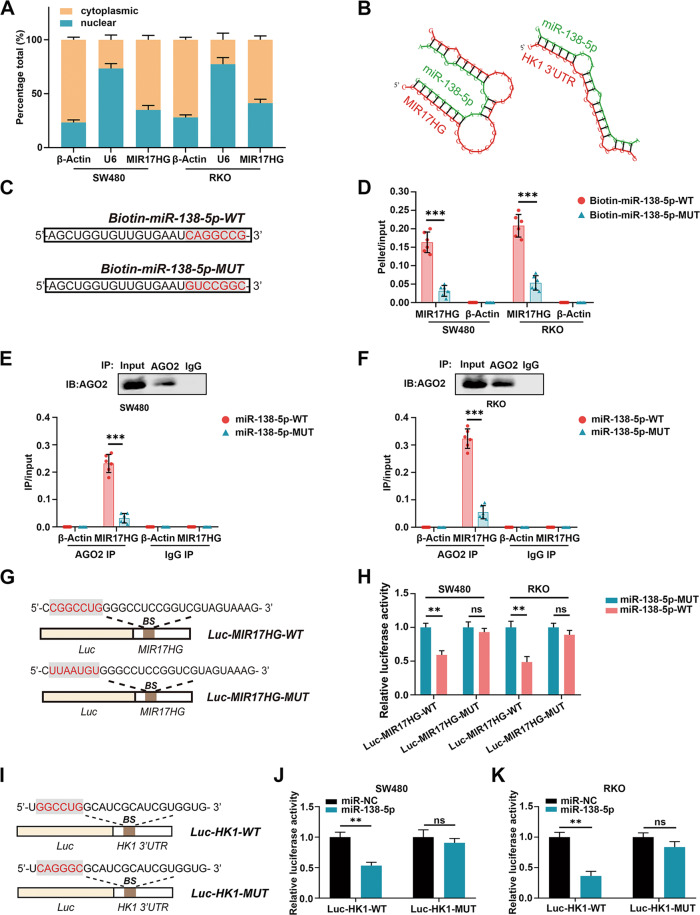
MIR17HG sponges miR-138-5p to upregulate HK1 expression in colorectal cancer cells. **A** The cytoplasmic and nuclear distribution of MIR17HG in colorectal cancer cells was measured by quantitative RT-PCR; **B** The binding sites in miR-138-5p, MIR17HG and the HK1 3′UTR were predicted by RNAhybrid; **C** The sequences of wild-type (WT) miR-138-5p and the designed mutant (MUT); **D** Levels of MIR17HG and β-Actin mRNA after streptavidin capture were measured in colorectal cancer cells transfected with wild-type biotinylated miR-138-5p or its mutant; **E**, **F** SW480 (**E**) and RKO (**F**) cells were transfected with wild-type miR-138-5p or the mutant. After AGO2 immunoprecipitation and validation of the efficiency by western blot analysis, the levels of MIR17HG and β-Actin mRNA were quantified by quantitative RT-PCR, and immunoprecipitate (IP)/input ratios were compared; **G** The sequences of reporter genes containing wild-type MIR17HG or its mutant without the miR-138-5p binding site (BS); **H** Effects of overexpression of wild-type miR-138-5p or its mutant on luciferase activity of wild-type or mutated MIR17HG in SW480 and RKO cells; **I** The sequences of reporter genes containing the wild-type HK1 3′ UTR or its mutant without the miR-138-5p binding site (BS); **J**, **K** Effects of miR-138-5p or the negative control miRNA on the luciferase activity of the wild-type or mutated HK1 3′ UTR in SW480 (**J**) and RKO (**K**) cells. **P < 0.01; ***P < 0.001; ns not significant.

As the roles of lncRNAs vary according to their subcellular localization, we further analyzed the distribution of MIR17HG in CRC cells using β-Actin as the cytoplasmic control and U6 as the nuclear control. The results showed that MIR17HG was present in both the cytoplasm and nucleus but was more concentrated in the cytoplasm (Fig. 5A). It has been reported that cytoplasmic lncRNA frequently functions as a competing endogenous RNA (ceRNA) to decoy miRNAs [21], whether MIR17HG can upregulate HK1 mRNA expression by acting as a miRNA sponge in CRC cells is unclear. A total of 13 candidate miRNAs targeting the 3′UTR of HK1 mRNA with high predictive scores as evaluated with online prediction software (TargetScan and miRanda) were selected. To investigate whether these candidate miRNAs can interact with MIR17HG, a plasmid containing a luciferase reporter gene with a MIR17HG fragment inserted at its downstream end (Luc-MIR17HG-WT) was designed and cotransfected with miRNA mimics into the indicated cells. Among these miRNAs, miR-138-5p showed the most significant inhibition of luciferase activity in both SW480 and RKO cells (Supplementary Fig. S4a). To verify whether MIR17HG sponged miR-138-5p, we simulated their complementary binding by using the online engine RNAhybrid. The results showed that MIR17HG and the 3′UTR of HK1 mRNA potentially shared the same miR-138-5p-binding sequence (Fig. 5B). Next, we designed biotinylated miR-138-5p mimics and constructed the corresponding mutant plasmids (Fig. 5C), which were transfected into SW480 and RKO cells for a biotin pulldown assay. MIR17HG was more highly enriched in the precipitate of wild-type biotin-coupled miR-138-5p than in that of the corresponding mutant (Fig. 5D), suggesting that MIR17HG is able to capture miR-138-5p via base pairing. As lncRNA is reported to sponge miRNA in an argonaute 2 (AGO2)-dependent manner [22], enrichment of MIR17HG was evaluated by qPCR after AGO2 immunoprecipitation in CRC cells transfected with wild-type or mutated miR-138-5p mimics. We found higher enrichment of MIR17HG in wild-type miR-138-5p-transfected cells, which implied that AGO2 could provide a platform for the interaction between MIR17HG and miR-138-5p (Fig. 5E, F). For further verification, we constructed a mutant MIR17HG fragment without the indicated miR-138-5p complementary sequence and inserted it downstream of the luciferase reporter gene (Luc-MIR17HG-MUT, Fig. 5G). In Luc-MIR17HG-WT-transfected cells, wild-type miR-138-5p markedly decreased luciferase activity compared with that in the mutant group. However, this effect was abolished in cells transfected with Luc-MIR17HG-MUT (Fig. 5H). These data indicated that MIR17HG functioned as a miR-138-5p sponge in CRC cells.

Furthermore, transfection of miR-138-5p mimics or inhibitors dramatically weakened the effects of MIR17HG upregulation or downregulation on both the mRNA and protein levels of HK1 (Supplementary Fig. S4b-e). To confirm the direct interaction at the complementary binding site in the 3′UTR of HK1 mRNA and miR-138-5p, we then constructed luciferase reporter vectors containing the wild-type 3′UTR of HK1 mRNA (Luc-HK1-WT) or a mutated 3′UTR sequence without the miR-138-5p complementary binding site (Luc-HK1-MUT, Fig. 5I). We found that transfection of miR-138-5p mimics sharply decreased luciferase activity compared with that in the negative control group of Luc-HK1-WT-transfected CRC cells (Fig. 5J, K). However, transfection of miR-138-5p mimics did not affect luciferase activity in Luc-HK1-MUT-transfected CRC cells (Fig. 5J, K). These results indicated that miR-138-5p suppressed HK1 expression by binding to its 3′UTR; however, this suppression was blocked by MIR17HG. Taken together, these findings indicate that MIR17HG functions as a ceRNA to promote HK1 expression by sponging miR-138-5p in CRC cells.

### MIR17HG sponges miR-138-5p to promote glycolysis in CRC cells

**Fig. 6:**
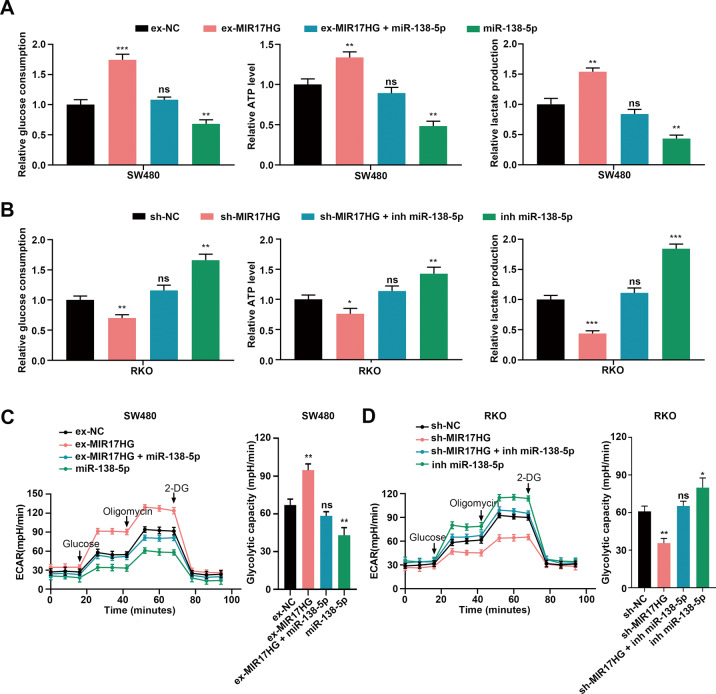
MIR17HG sponges miR-138-5p to promote glycolysis in CRC cells. **A**, **B** Relative changes in glucose consumption, ATP levels and lactate production induced by transfection of miR-138-5p mimics into SW480 cells with or without MIR17HG overexpression (**A**) and transfection of miR-138-5p inhibitors into RKO cells with or without MIR17HG knockdown (**B**); **C**, **D** Effects of transfecting miR-138-5p mimics on ECAR levels in SW480 cells with or without MIR17HG overexpression (**C**) and effects of transfecting miR-138-5p inhibitors into RKO cells with or without MIR17HG knockdown (**D**). *P < 0.05; **P < 0.01; ***P < 0.001; ns not significant.

We further explored whether MIR17HG could also enhance glycolysis in CRC cells by sponging miR-138-5p. Then, we transfected miR-138-5p mimics or inhibitors into SW480/HT29 cells with MIR17HG overexpression or RKO/SW620 cells with MIR17HG knockdown, respectively, and into their corresponding control cells. We found that glucose uptake, lactate production, and ATP production were significantly decreased or increased after transfection of miR-138-5p mimics or miR-138-5p inhibitors into MIR17HG-overexpressing or knockdown CRC cells, respectively (Fig. 6A, B, Supplementary Fig. S5a-b). The alterations in glycolytic capacity were consistent with the above findings (Fig. 6C, D, Supplementary Fig. S5c-d). MIR17HG, also known as miR-17~92 cluster gene, could promote miR-17, miR-18a, miR-19a, miR-19b-1, miR-20a, and miR-92a-1 expression. However, we did not find miR-17~92 cluster could affect CRLM via regulating glycolysis (Supplementary Fig. S6a-e). These findings indicated that MIR17HG actually promoted glycolysis by sponging miR-138-5p in CRC cells.

### MIR17HG functions as a ceRNA to promote the invasion and liver metastasis of CRC cells in vitro and in vivo

**Fig. 7:**
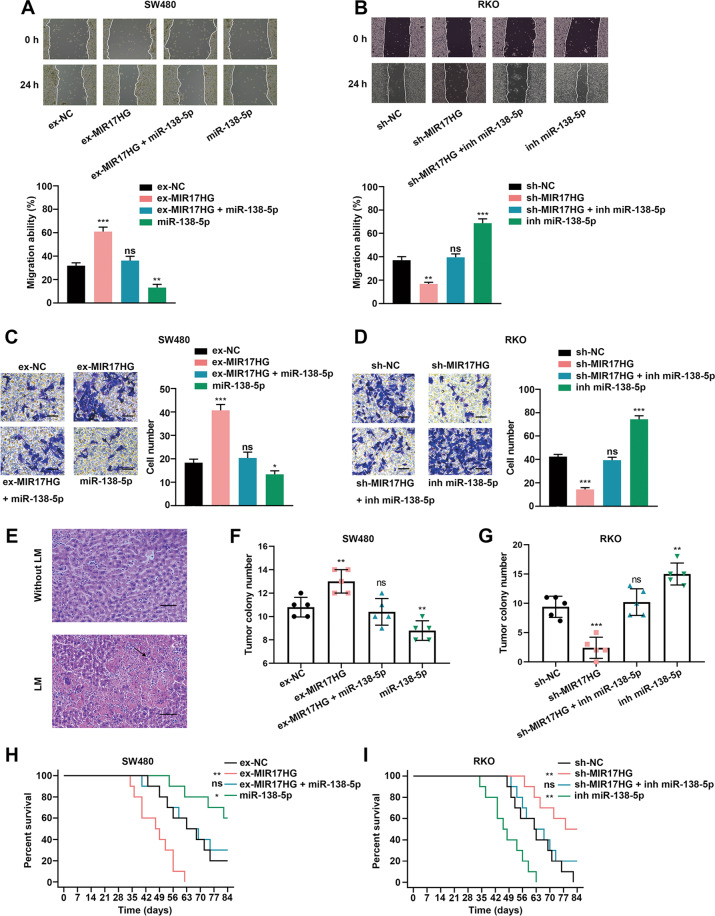
MIR17HG promotes the invasion and liver metastasis of colorectal cancer cells by sponging miR-138-5p in vitro and in vivo. **A**, **B** A wound healing assay was used to evaluate the effects of miR-138-5p mimics or inhibitors on the migration ability of SW480 (**A**) and RKO (**B**) cells upon MIR17HG overexpression and knockdown and in the corresponding control cells; **C**, **D** A transwell assay was applied to evaluate the effects of miR-138-5p mimics or inhibitors on the invasive ability of SW480 cells (**C**) or RKO cells with MIR17HG overexpression or knockdown and their corresponding control cells (**D**); **E** Representative HE images of liver tissues obtained from nude mice with (lower panel) or without (upper panel) liver metastasis, original magnification, ×200; scale bar, 50 μm; **F**,**G** The number of liver metastatic foci in mice of each group (*n* = 5 for each group) was counted under a microscope; **H**, **I** Overall survival of each group of mice injected with engineered SW480 (**H**) or RKO (**I**) cells (*n* = 10 for each group). *P < 0.05; **P < 0.01; ***P < 0.001; ns not significant.

Furthermore, we performed in vitro wound healing assays and transwell assays to evaluate the effects of MIR17HG sponging miR-138-5p. We found that transfection of miR-138-5p mimics into MIR17HG-overexpressing CRC cells or miR-138-5p inhibitors into MIR17HG-knockdown CRC cells weakened the promoting or inhibitory effects of MIR17HG overexpression or knockdown, respectively, on the wound healing ability (Fig. 7A, B, Supplementary Fig. S7a-b). In addition, transwell assay showed that transfection of miR-138-5p mimics or inhibitors reduced or increased the number of invaded CRC cells upon overexpression or knockdown of MIR17HG, respectively (Fig. 7C, D, Supplementary Fig. S7c-d). Accordingly, these data indicated that MIR17HG promoted the migration and invasion of CRC cells by sponging miR-138-5p in vitro.

Next, we also performed in vivo liver metastasis assay to investigate whether MIR17HG affects liver metastasis in mice by sponging miR-138-5p via injecting CRC cells from all experimental and control groups into spleens of nude mice. HE staining was performed to count the number of metastatic colonies per field of view in the livers of mice. We found that injection of MIR17HG-overexpressing CRC cells transfected with miR-138-5p mimics or MIR17HG-knockdown CRC cells transfected with miR-138-5p inhibitors partially reduced or increased, respectively, the number of liver metastatic colonies in mice compared with those in control groups (Fig. 7E-G, Supplementary Fig. S7e-f). Moreover, the reduced OS time of mice in MIR17HG overexpression group was totally offset by cotransfection of miR-138-5p mimics, while the increased OS time in MIR17HG knockdown group was counteracted by cotransfection of miR-138-5p inhibitors (Fig. 7H, I). Importantly, our clinical data analyses showed that miR-138-5p expression was lower in patients with liver metastasis than in those without and that low miR-138-5p expression in tumor tissues of CRC patients predicted poor OS, especially in patients with liver metastasis (Supplementary Fig. S8a-d). In addition, there was an inverse correlation between miR-138-5p and MIR17HG expression (Supplementary Fig. S8e), and the level of miR-138-5p was negatively correlated with the IHC staining score of HK1 in 105 CRC tissues (Supplementary Fig. S8f). And a low miR-138-5p level was positively correlated with distant metastasis and advanced stage of CRC (Supplementary Table S10). In addition, the levels of miR-138-5p were gradually decreased, while mRNA and protein levels of HK1 were gradually increased in adjacent normal, CRC and paired liver metastatic tissues used for RNA-seq (Supplementary Fig. S8g-j). On the other hand, low miR-138-5p expression predicted poor recurrence-free survival in CRC patients based on TCGA dataset (Supplementary Fig. S8k). Notably, GEPIA public dataset (http://gepia.cancer-pku.cn/) analysis showed that the mRNA level of HK1 had no difference between normal and CRC tissues, and also no effects on recurrence-free survival of CRC patients (Supplementary Fig. S8l). According to another report that HK1 protein was markedly upregulated in primary and metastatic CRC tissues relative to normal mucosa [23], HK1 mainly plays its carcinogenesis role at the translation rather than transcription level. Taken together, these results revealed that MIR17HG functioned as a ceRNA by sponging miR-138-5p to upregulate HK1 levels and drive CRLM.

### Accumulated lactate upregulates MIR17HG expression via the p38/Elk-1 pathway in a feedback loop

**Fig. 8:**
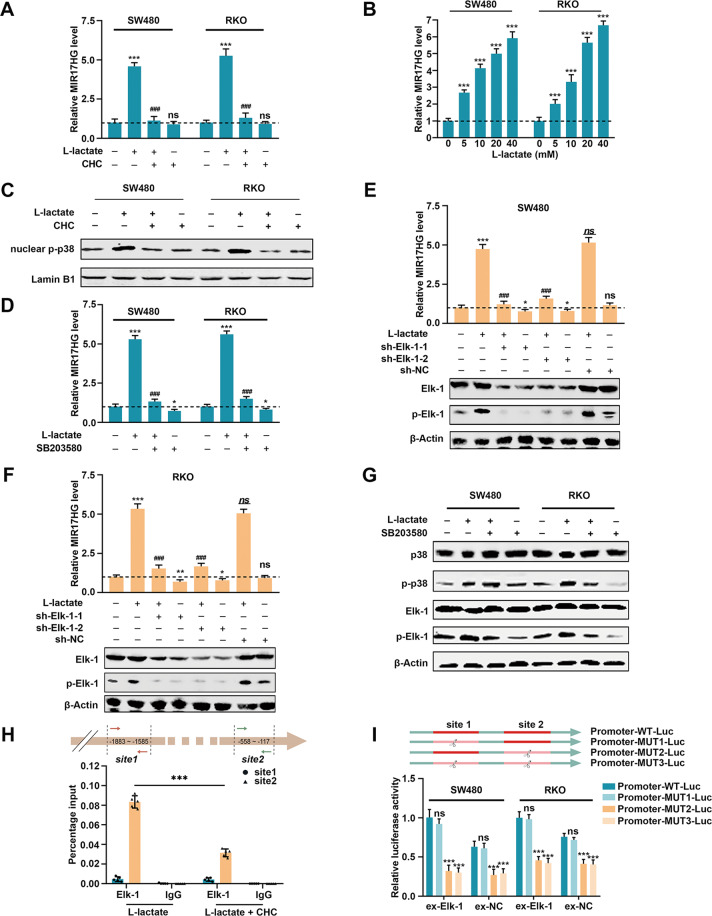
Lactate accumulation upregulates MIR17HG transcriptional expression via the p38/Elk-1 pathway. **A** Effects of the monocarboxylate transporter inhibitor CHC (5 mM) on MIR17HG levels in SW480 and RKO cells stimulated with lactate (20 mM) for 12 h; **B** Different concentrations of L-lactate were used to stimulate SW480 and RKO cells for 12 h prior to quantitative determination of MIR17HG expression; **C** Nuclear translocation of phosphorylated p38 in lactate-stimulated cells was verified by western blot analysis; **D** Effects of the p38 chemical inhibitor SB203580 (10 μM for 12 h) on MIR17HG transcription in SW480 and RKO cells stimulated with lactate; **E**, **F** MIR17HG levels were detected after transfection of sh-Elk-1 plasmids into SW480 (**E**) and RKO (**F**) cells with or without lactate stimulation; **G** Effects of lactate and SB203580 on activation of p38 and Elk-1 in SW480 and RKO cells; **H** Schematic illustration of the binding site of Elk-1 in the MIR17HG promoter (upper panel); a ChIP assay was performed to investigate the roles of the two Elk-1 binding sites on the transcriptional activity of the MIR17HG promoter (lower panel); **I** Schematic illustration of reporter genes with the wild-type promoter of MIR17HG and its three mutants (upper panel); four kinds of reporter gene vectors were transfected into SW480 or RKO cells stimulated with lactate to confirm the binding site of activated Elk-1 in the MIR17HG promoter (lower panel). *P < 0.05; **P < 0.01; ***P < 0.001; ^###^*P* < 0.001; ns and *ns* not significant. *, **, *** and ns indicate the statistical difference between the corresponding group and the group with no administration in Fig. 8A, D, E and F. ^###^ and *ns* indicate the statistical difference between the corresponding group and the group stimulated by lactate only.

More interestingly, we used α-cyano-4-hydroxycinnamate (CHC, Fig. 8A), a monocarboxylate transporter inhibitor [24, 25], to block lactate import into cells and found that MIR17HG was also downregulated, which indicated that lactate accumulated via glycolysis might regulate MIR17HG expression in return. Subsequently, SW480 and RKO cells were incubated with different concentrations of L-lactate, and we observed that lactate also significantly upregulated MIR17HG in a dose-dependent manner (Fig. 8B). These data further revealed that lactate-activated MIR17HG expression in CRC cells.

As NF-κB [26], Wnt [27], p38 [28], JNK [28], and ERK1/2-MAPK [29, 30] pathways have the potential to be activated by lactate, we next investigated the activation of these pathways to confirm the mechanism of MIR17HG upregulation in lactate-stimulated CRC cells. The luciferase reporter assays showed no changes in the NF-κB signaling pathways after lactate stimulation (Supplementary Fig. S9a-b). TOP/FOP flash reporter assay has been widely used to evaluate the activity of Wnt signaling pathway [31]. Thus, we also used this system to evaluate the activity of Wnt signaling pathway, however, there was no change upon lactate stimulation (Supplementary Fig. S9c-d). Notably, lactate accumulation increased the phosphorylation of p38 (p-p38) but not ERK1/2 or JNK, and CHC significantly abrogated the lactate-induced increase in p38 phosphorylation (Supplementary Fig. S9e). As the catalytic function of p38 requires nuclear translocation of its phosphorylated form, we next determined the nuclear level of phosphorylated p38 and found that lactate facilitated p38 phosphorylation and its nuclear translocation (Fig. 8C). To further confirm whether lactate upregulates MIR17HG transcription via the p38-MAPK pathway, we added SB203580, a canonical inhibitor proven to block the activity of p38 kinase, into the culture medium [32, 33]. We found that SB203580 partially suppressed the lactate-induced MIR17HG expression (Fig. 8D). Even though no lactate incubation was performed, we still discovered a suppressive effect of SB203580 on MIR17HG transcription in SW480 and RKO cells (Fig. 8D). Accordingly, the lactate-activated p38-MAPK signaling pathway contributed to MIR17HG upregulation in CRC cells.

Subsequently, to predict p38 downstream transcription factors, we screened the MIR17HG promoter sequence from −2000 bp upstream to +100 bp downstream of the transcription start site using the PROMO engine, with a dissimilarity rate of <3%. Then, we knocked down a total of eight candidate transcription factors and found that silencing of only Elk-1 downregulated MIR17HG expression and significantly suppressed lactate-induced MIR17HG expression (Fig. 8E, F, Supplementary Fig. S9f-g). We further discovered that lactate enhanced the phosphorylation of Elk-1, which was completely abolished by SB203580-induced inhibition of p38 (Fig. 8G). However, the level of phosphorylated p38 did not seem to be disturbed by SB203580 in SW480 and RKO cells (Fig. 8G), probably because SB203580 mainly inhibits the catalytic ability of p38 to phosphorylate its substrates but not phosphorylation of p38 itself [34]. These results clarified that lactate could activate the p38/Elk-1 pathway to promote MIR17HG transcription in CRC cells. Furthermore, to examine the effect of Elk-1 on MIR17HG transcription, we predicted two high-confidence potential binding sites for Elk-1 in the promoter of MIR17HG, then chromatin immunoprecipitation (ChIP) assay revealed that lactate significantly promoted the enrichment of Elk-1 at site 2 but not site 1 in the MIR17HG promoter (Fig. 8H). Furthermore, we synthesized three truncation mutants of the MIR17HG promoter without site 1 (Promoter-MUT1-Luc), site 2 (Promoter-MUT2-Luc) or both sites (Promoter-MUT3-Luc), which were cloned into the pGL4.20 vector and transfected into Elk-1-overexpressing SW480 /RKO cells upon lactate stimulation (Fig. 8I). The markedly decreased luciferase activity in Promoter-MUT2-Luc- and Promoter-MUT3-Luc-transfected cells indicated that Elk-1 only controlled MIR17HG transcription by binding to site 2 upon lactate accumulation (Fig. 8I). Taken together, these data demonstrated that lactate accumulation activating the p38/Elk-1 signaling pathway promoted the transcriptional expression of MIR17HG in CRC cells, forming a positive feedback loop that resulted in persistent aerobic glycolysis and in CRLM.

## Discussion

CRLM is one of the main causes of death in CRC patients [4]. Insufficient knowledge of the mechanism concerning CRLM limits the efficacy of treatment and results in poor survival. It is well established that aerobic glycolysis plays significant roles in reprogramming tumor metastatic microenvironment [9, 35]. The present study identified that glycolysis-related lncRNA MIR17HG enhanced aerobic glycolysis and promoted liver metastasis. More interestingly, lactate accumulated via glycolysis also upregulated MIR17HG expression via the p38/Elk-1 pathway in return, forming a positive feedback loop between MIR17HG expression and glycolysis and eventually resulting in CRLM (Supplementary Fig. S10). These findings revealed a novel interaction mechanism among lncRNA MIR17HG, glycolysis, and metastatic microenvironment, which might be helpful to develop new strategies for predicting the risk of CRLM and designing treatments in the future.

Reportedly, MIR17HG is able to interact with silent information regulator 1, a member of the histone deacetylase family, for DNA damage repair [36]. In addition, MIR17HG might exert its antitumor effect by decreasing the methylation level of miR-142-3p in non-small cell lung cancer [37]. MIR17HG could also promoted CRC initiation and development via activating NF-κB pathway and PD-L1-induced immunosuppression [38]. Thus, MIR17HG performed oncogenic or antitumor functions in different kinds of tumors. Our present study first showed that MIR17HG enhanced glycolysis in CRC cells, which supplemented the roles of MIR17HG in cancers. Mechanically, as the first rate-limiting enzyme of glycolysis, hexokinase (HK) family members function as the gatekeeper of energy flux through the glycolytic pathway. HK2 is the most investigated HK family member and has been identified as a tumor promoter in various cancers, such as gallbladder, liver, and prostate cancer [8, 39, 40]. However, we found that HK1, another member of the HK family, was upregulated by MIR17HG in CRC cells, consistent with a previous report that HK1 was markedly upregulated in metastatic CRC tissues [23]. These data not only revealed the specific target of MIR17HG-regulated glycolysis during CRLM but also showed that the HK family might play different roles in various cancers. In cytoplasm, lncRNAs frequently sponge miRNAs to block miRNA-induced gene silencing [13, 14]. We found cytoplasmic enrichment of MIR17HG in CRC cells. Subsequent series of assays demonstrated that MIR17HG sponged miR-138-5p to upregulate HK1, leading to enhanced glycolysis and malignancy both in vitro and in vivo. Therefore, combined evaluation of MIR17HG, miR-138-5p and HK1 might provide an efficient prognostic indicator in patients with CRLM.

More interestingly, feedback upregulation of MIR17HG by enhanced glycolysis was observed in the present study. Recently, the glycolytic metabolite lactate has been widely demonstrated to reprogram tumor metastatic microenvironment [28]. We also found that lactate upregulated MIR17HG in CRC cell lines in a dose-dependent manner and that this upregulation was abolished by the lactate transporter blocker CHC. Mechanistically, we demonstrated that lactate upregulated MIR17HG and formed a feedback loop by activating the p38-MAPK/Elk-1 axis. Although the ERK-MAPK signaling pathway has been reported to be activated by lactate [29] and lactate has been demonstrated to promote the expression of the lncRNA HISLA via the ERK pathway [30], our study suggested a novel role of p38 in regulating lncRNA expression and CRLM, which revealed a new mechanism of the p38-MAPK signaling pathway and glycolysis. However, it is unclear whether glycolysis-accumulated lactate directly activates p38. Moreover, whether other glycolytic metabolites are also involved in this positive feedback loop needs further exploration.

In conclusion, our study revealed that glycolysis-related lncRNA MIR17HG functioned as a ceRNA in promoting CRC cell glycolysis and CRLM; moreover, the accumulated lactate could result in MIR17HG expression via the p38/Elk-1 pathway, forming a positive feedback loop between glycolysis and MIR17HG. Our findings may provide novel strategies for predicting the risk of CRLM as well as new insights into understanding the tumor metastasis-associated lactate microenvironment and the development of therapeutics for metastatic CRC.

## Materials and methods

### Quantitative real-time PCR

RNA was obtained using TRIzol reagent (Beyotime Biotechnology, Shanghai, China) and reverse transcribed into cDNA. Quantitative real-time PCR was performed using SYBR qPCR Master Mix (Vazyme, Nanjing, China) in an ABI 7500-Fast Real-Time PCR System (SeqGen, Los Angeles, CA, USA). Expression levels were normalized to those of the appropriate internal control (β-Actin or U6) and are presented as the mean ± standard deviation (SD) of at least three independent experiments. Primer sequences are reported in Supplementary Table S11.

### Western blotting

Proteins were obtained by using total protein or nuclear protein extraction kits (Beyotime Biotechnology, Shanghai, China). Membranes were incubated with primary antibodies overnight at 4 °C and then with horseradish peroxidase-conjugated secondary antibodies for 1 h. Signals were visualized using ECL reagent (Pierce, Rockford, IL, USA). β-Actin and Lamin B1 were used as internal controls. Details of the primary antibodies are listed in Supplementary Table S12.

Other assays used in this study are described in ***Supplementary Information***.

## Supplementary information

Supplementary Information

Supplementary Table S1

Supplementary Table S2

Supplementary Table S3

Supplementary Table S4

Supplementary Table S5

Supplementary Table S6

Supplementary Table S7

Supplementary Table S8

Supplementary Table S9

Supplementary Table S10

Supplementary Table S11

Supplementary Table S12

Description of Supplementary Materials
