# Supplementary material for: LncRNA MIR17HG promotes colorectal cancer liver metastasis by mediating a glycolysis-associated positive feedback circuit
Source: Oncogene. 2021 Jun 18;40(28):4709–24. doi: 10.1038/s41388-021-01859-6 (PMC8282501; doi:10.1038/s41388-021-01859-6)
Supplement: Supplementary file 1 — Supplementary Information [file 41388_2021_1859_MOESM1_ESM.pdf]

Supplementary Fig. S1

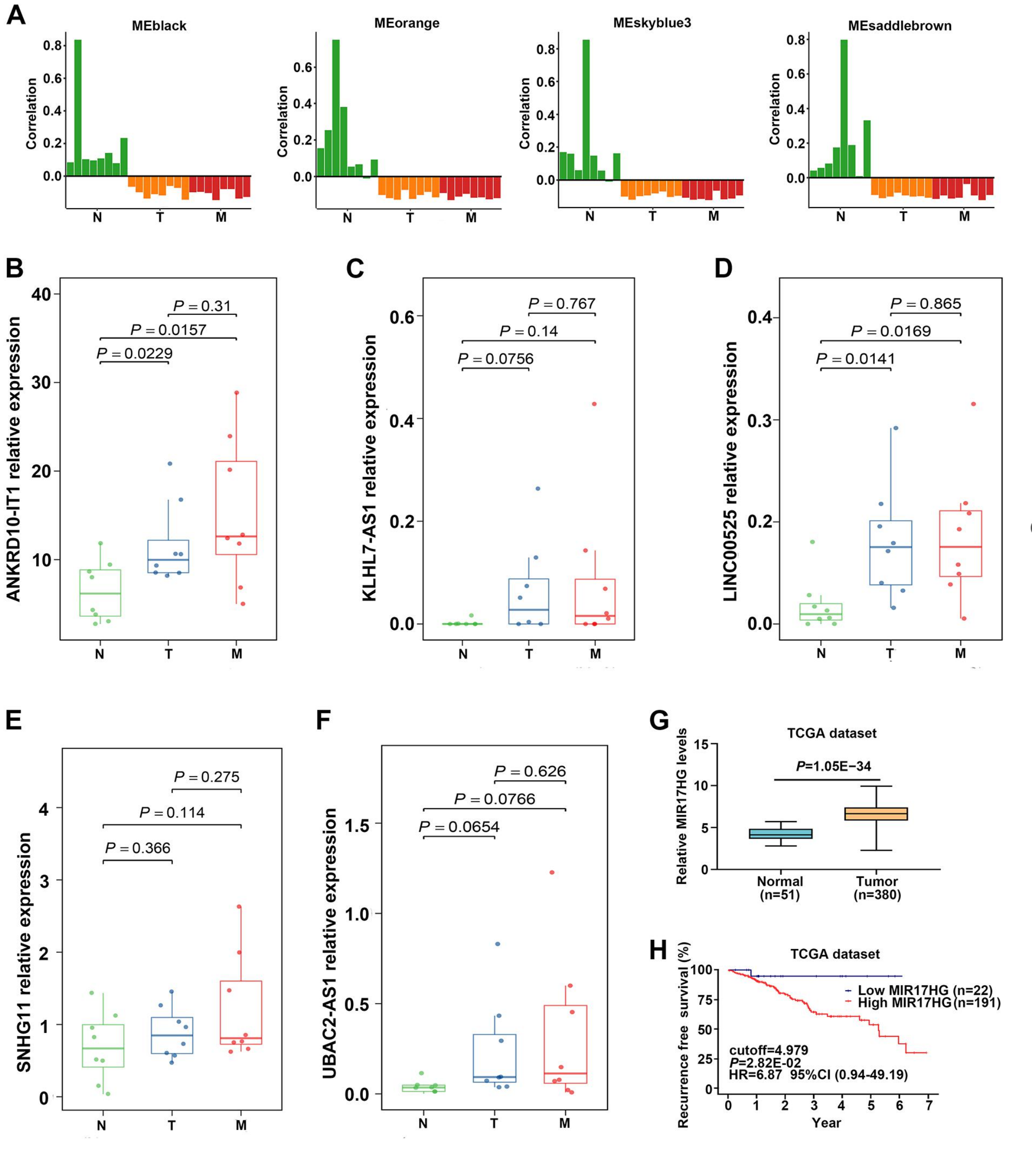

Supplementary Fig. S2

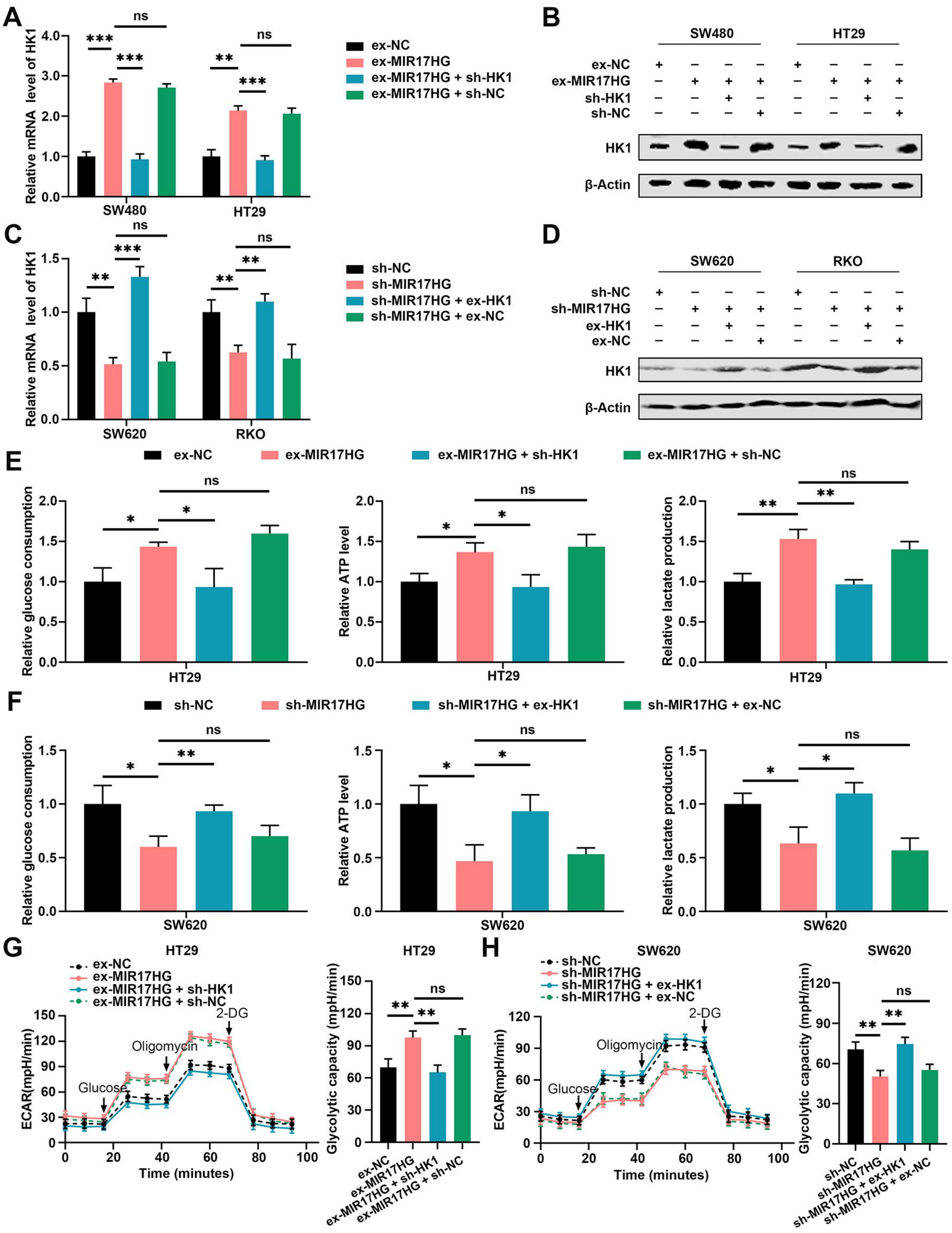

Supplementary Fig. S3

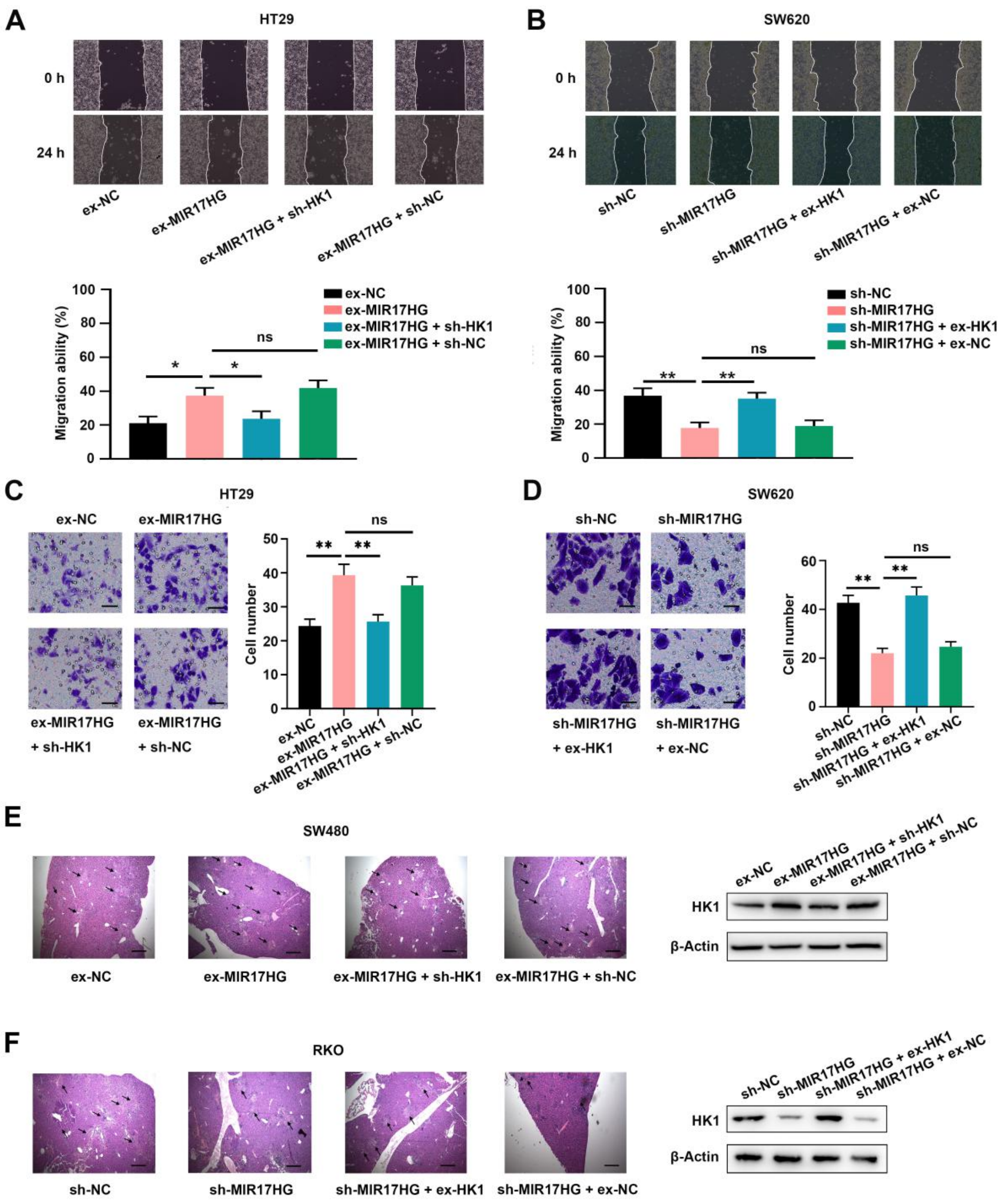

Supplementary Fig. S4

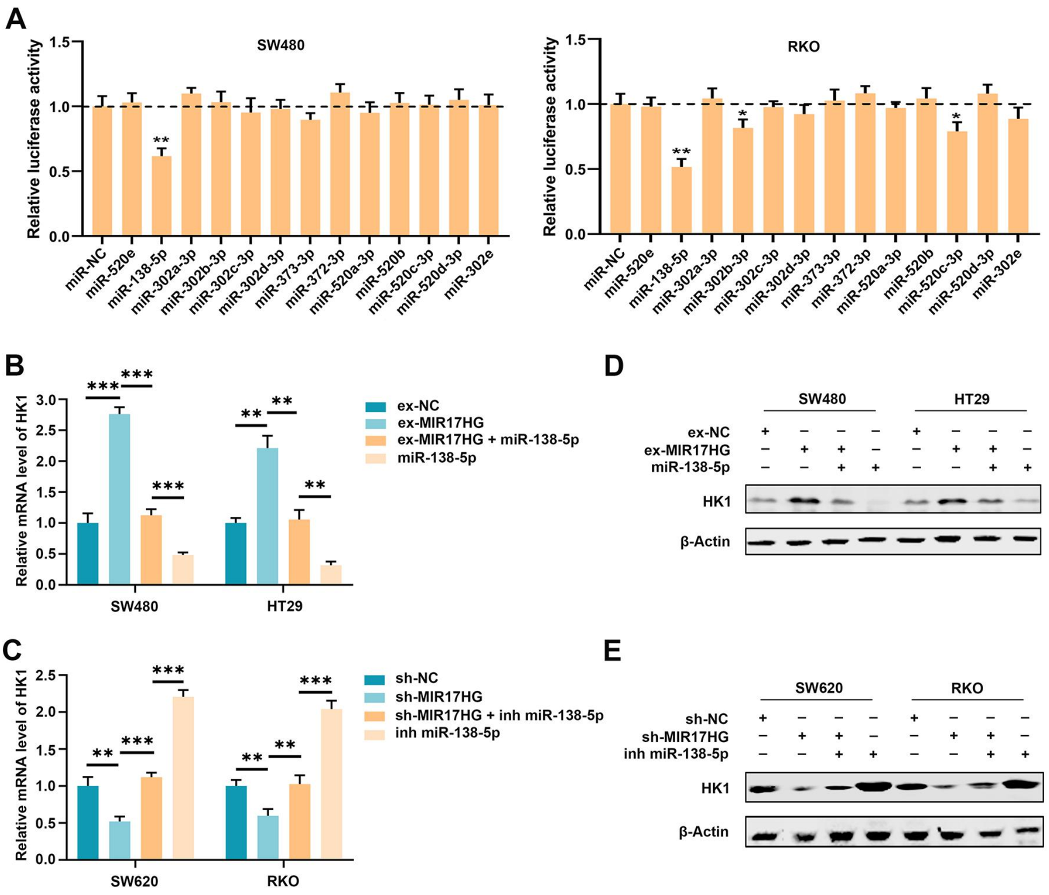

Supplementary Fig. S5

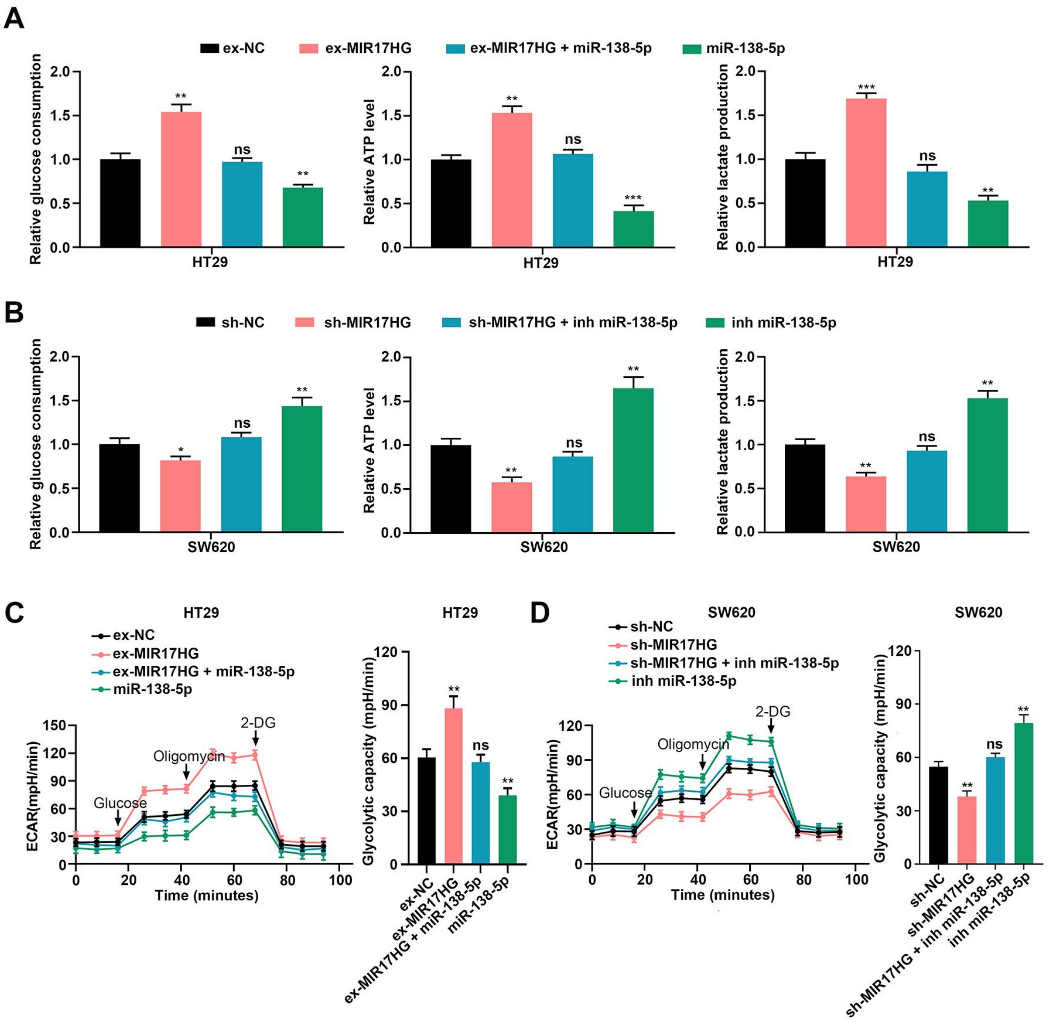

Supplementary Fig. S6

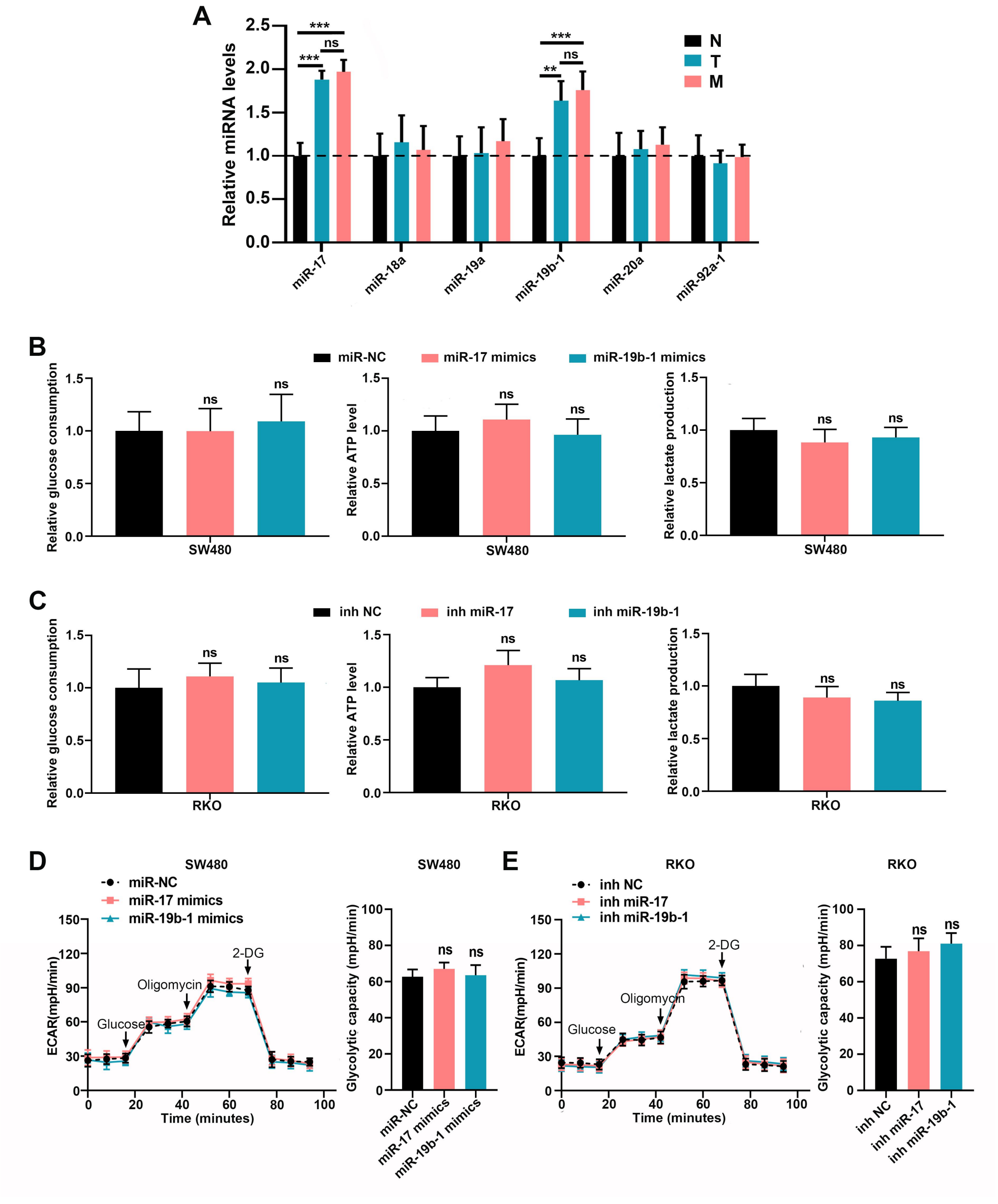

Supplementary Fig. S7

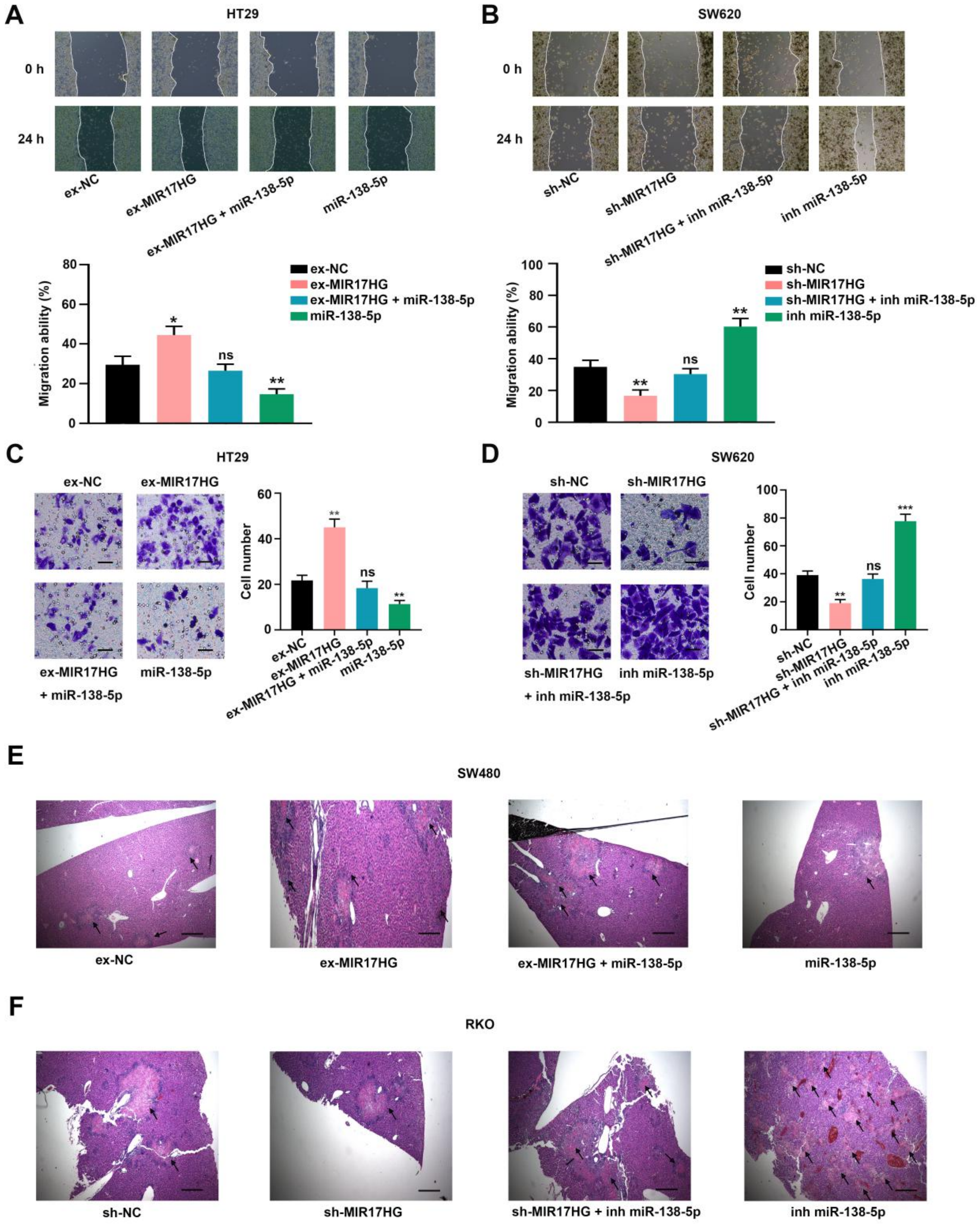

Supplementary Fig. S8

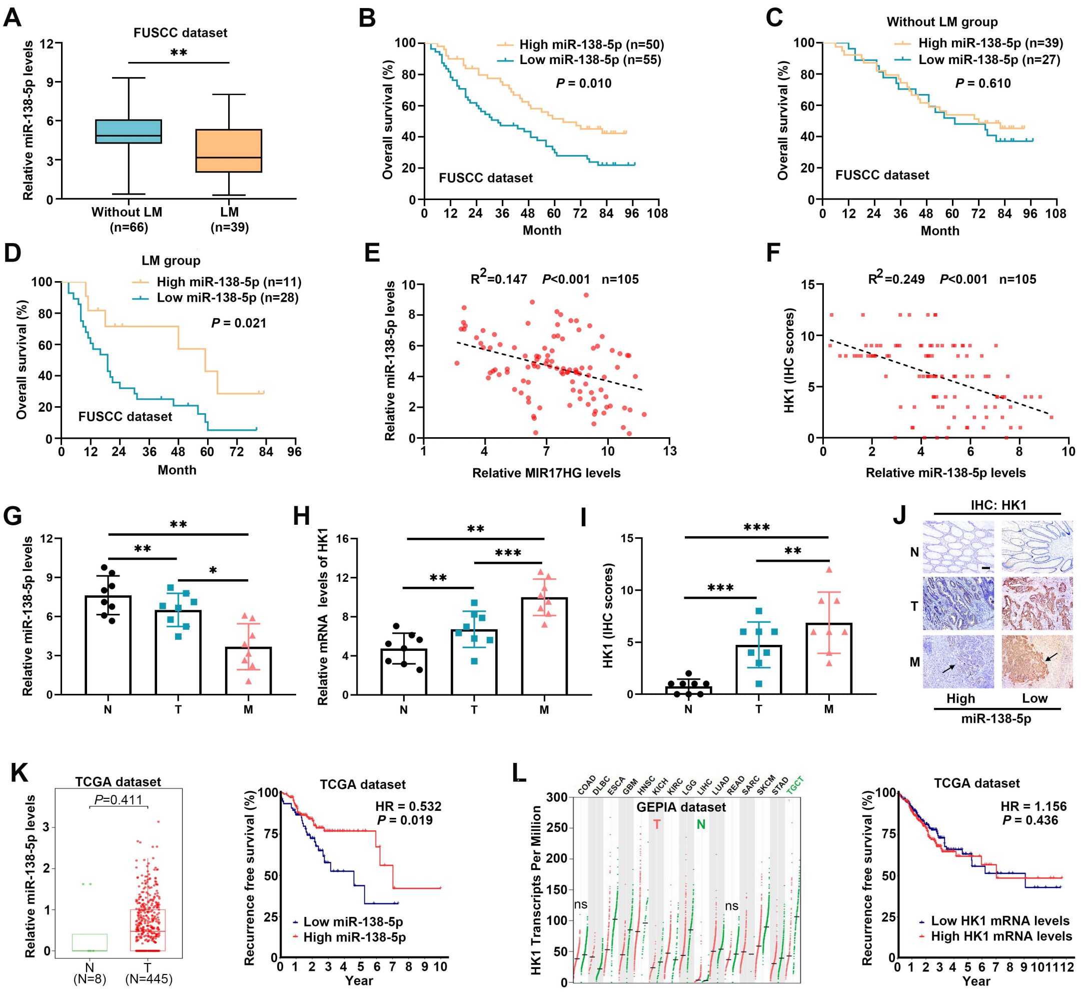

Supplementary Fig. S9

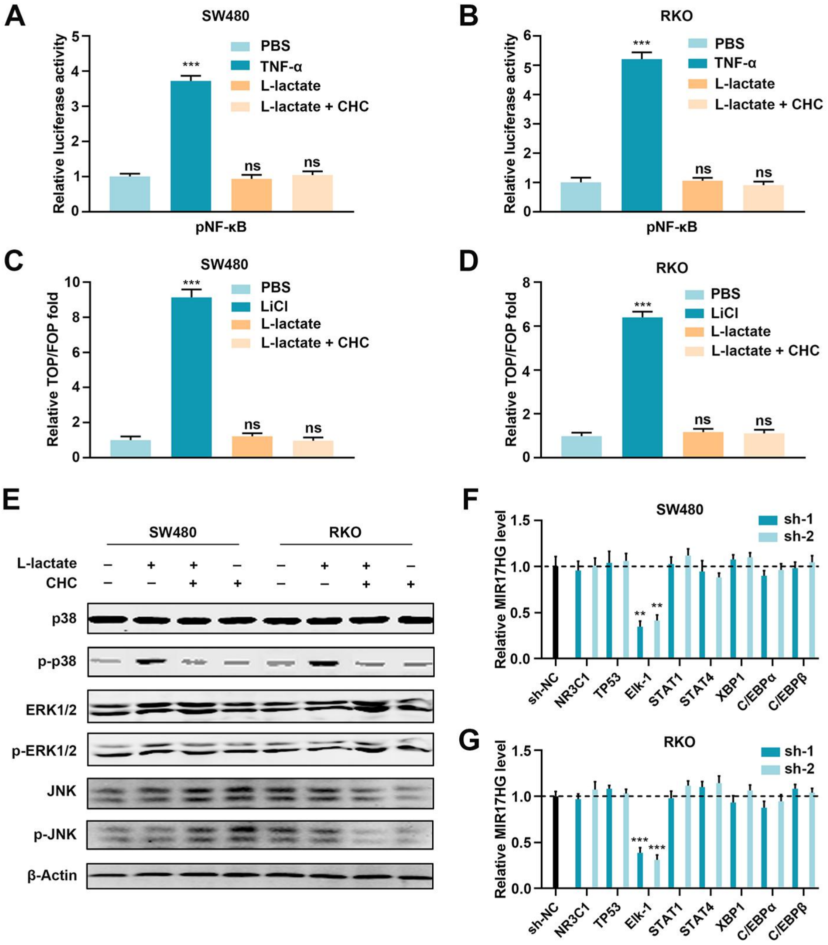

Supplementary Fig. S10

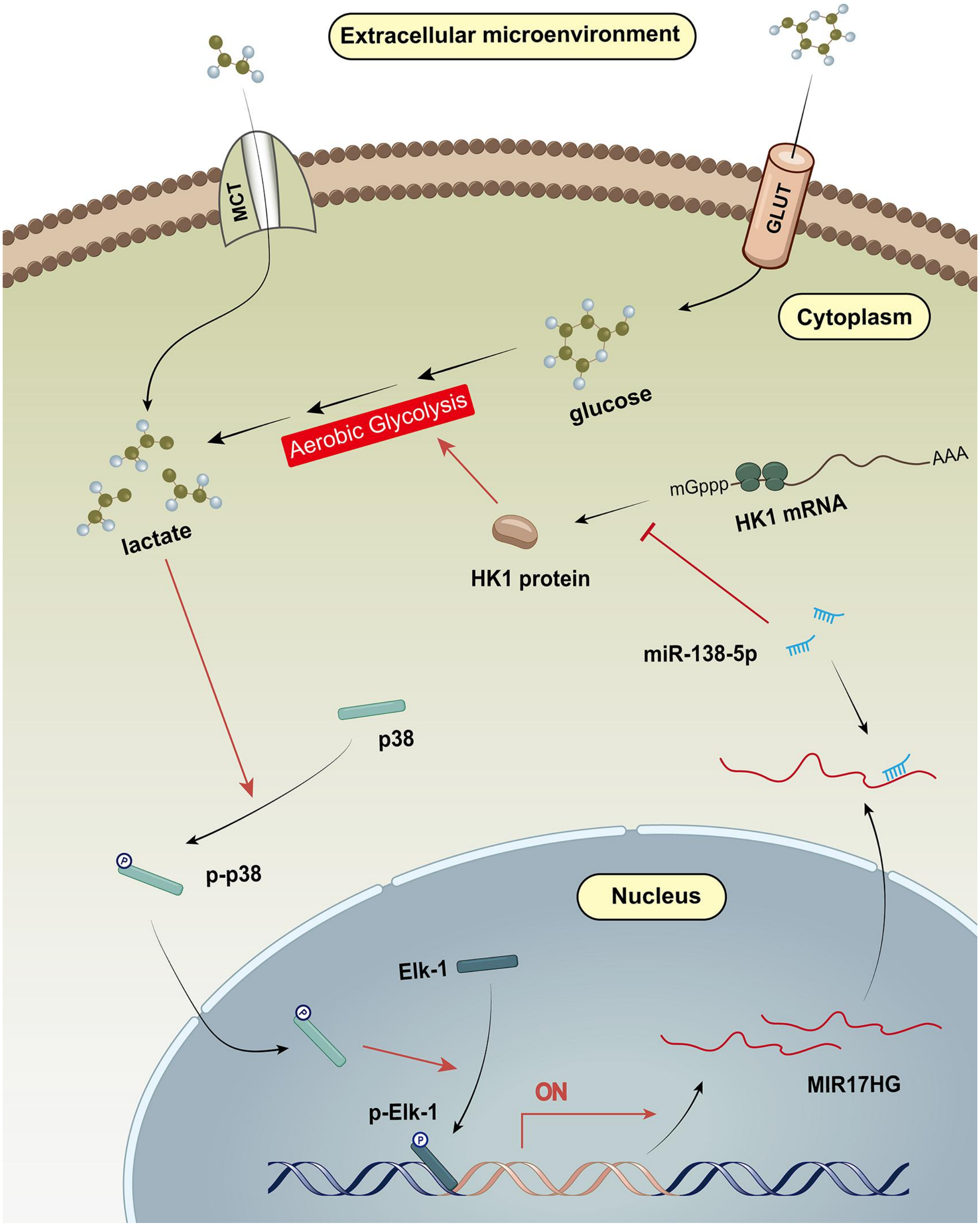

Supplementary Fig. S1. Comprehensive WGCNA identified dysregulated genes involved in colorectal cancer liver metastasis. a. Four modules showed decreases from adjacent normal to CRC to paired liver metastatic tissues, while no significant difference was found between the groups of CRC and paired liver metastatic tissues; b, c, d, e, f. Expression levels of another five dysregulated lncRNAs in the comprehensive network in adjacent normal, CRC and paired liver metastatic tissues; g. The expression levels of MIR17HG in 51 normal tissues and 380 colorectal cancer tissues in the TCGA database; h. Kaplan-Meier analysis with the log-rank test for recurrence-free survival in 213 colorectal cancer patients from TCGA database according to MIR17HG expression.

Supplementary Fig. S2. MIR17HG promotes glycolysis in colorectal cancer cells in an HK1-dependent manner. a, b. mRNA (a) and protein (b) levels of HK1 in MIR17HG-overexpressing SW480 and HT29 cells with or without HK1 knockdown were determined by quantitative RT-PCR and western blotting, respectively; c, d. mRNA (c) and protein (d) levels of HK1 in MIR17HG-knockdown SW620 and RKO cells with or without HK1 overexpression were analyzed by quantitative RT-PCR and western blotting, respectively; e, f. Relative levels of glucose consumption, ATP production and lactate production in MIR17HG-overexpressing HT29 cells with or without HK1 knockdown (e) and MIR17HG-knockdown SW620 cells with or without HK1 overexpression (f); g, h. ECAR levels in MIR17HG-overexpressing HT29 cells with or without HK1 knockdown (g) and MIR17HG-knockdown SW620 cells with or without HK1 overexpression (h). *$P < 0.05$; **$P < 0.01$; ***$P < 0.001$; ns, not significant.

Supplementary Fig. S3. MIR17HG fostered the invasion and liver metastasis of CRC cells in

vitro and in vivo by upregulating HK1 expression. a, b. Effects of knockdown or overexpression of HK1 on the migration ability of MIR17HG-overexpressing HT29 cells (a) and MIR17HG-knockdown SW620 cells (b) were evaluated by a wound healing assay; c, d. Effects of HK1 knockdown or overexpression on the invasive ability of MIR17HG-overexpressing HT29 cells (c) and MIR17HG-knockdown SW620 cells (d) were evaluated by a transwell assay; e, f. Representative HE images of liver tissues and HK1 protein leveles on metastatic lesions obtained from nude mice injected with all variants of engineered SW480 (e) and RKO (f) cells; original magnification, 40 ×; scale bar, 50 μm. *$P < 0.05$; **$P < 0.01$; ***$P < 0.001$; ns, not significant.

Supplementary Fig. S4. MiR-138-5p impairs the effect of MIR17HG on HK1 expression. a. Effects of overexpression of 13 candidate miRNAs on the luciferase activity of wild-type MIR17HG in SW480 cells (left panel) and RKO cells (right panel); b. Effects of miR-138-5p mimics on HK1 mRNA levels in the indicated cells with or without MIR17HG overexpression; c. Effects of miR-138-5p inhibitors on HK1 mRNA levels in the indicated cells with or without MIR17HG knockdown; d. Effect of transfecting miR-138-5p mimics into SW480 and HT29 cells with or without MIR17HG overexpression on HK1 protein levels; e. Effect of transfecting miR-138-5p inhibitors into SW620 and RKO cells with or without MIR17HG knockdown on HK1 protein levels. *$P < 0.05$; **$P < 0.01$; ***$P < 0.001$; ns, not significant.

Supplementary Fig. S5. MIR17HG promoted glycolysis in CRC cells by sponging miR-138-5p. a, b. Relative changes in glucose consumption, ATP levels and lactate production after transfection of miR-138-5p mimics into HT29 cells with or without

MIR17HG overexpression (a) and upon transfection of miR-138-5p inhibitors into SW620 cells with or without MIR17HG knockdown (b); c, d. Effects of transfecting miR-138-5p mimics into HT29 cells with or without MIR17HG overexpression on ECAR levels (c) and transfecting miR-138-5p inhibitors into SW620 cells with or without MIR17HG knockdown (d). *P < 0.05; **P < 0.01; ***P < 0.001; ns, not significant.

Supplementary Fig. S6. Roles of MIR17HG-controlled miR-17~92 clusters on glycolysis of colorectal cancer cells. a. The expression levels of miR-17~92 cluster in adjacent normal, CRC and paired liver metastatic tissues; b. Changes of relative glucose consumption, ATP levels and lactate production in SW480 cells transfected with miR-17 or miR-19b-1 mimics; c. Changes of glucose consumption, ATP levels and lactate production in RKO cells upon miR-17 or miR-19b-1 inhibition; d. Changes of glycolytic capacity upon miR-17 or miR-19b-1 overexpression in SW480 cells; e. Changes of glycolytic capacity in miR-17- or miR-19b-1-inhibited RKO cells. **P < 0.01; ***P < 0.001; ns, not significant.

Supplementary Fig. S7. MIR17HG promotes the invasion and liver metastasis of CRC cells by sponging miR-138-5p in vitro and in vivo. a, b. A wound healing assay was used to evaluate the effects of miR-138-5p mimics or inhibitors on the migration ability of HT29 (a) or SW620 (b) cells with MIR17HG overexpression or knockdown and their corresponding control cells; c, d. A transwell assay was applied to evaluate the effects of miR-138-5p mimics or inhibitors on the invasive ability of HT29 cells (c) or SW620 cells with MIR17HG overexpression or knockdown and their corresponding control cells (d); e, f. Representative HE images of liver tissues obtained from nude mice injected with all variants of engineered SW480 (e) and RKO (f) cells; original magnification, 40 ×; scale bar, 50 μm. *P < 0.05; **P

< 0.01; ***P < 0.001; ns, not significant.

Supplementary Fig. S8. Low expression of miR-138-5p predicted poor survival in CRLM patients. a. The expression levels of miR-138-5p in 105 colorectal cancer tissues from patients without and with liver metastasis in the FUSCC dataset; b. Kaplan-Meier analysis with the log-rank test for overall survival of 105 colorectal cancer patients stratified by the miR-138-5p expression level; c, d. Kaplan-Meier analysis with the log-rank test for overall survival in the liver metastasis-free group (c) and liver metastasis group (d); e, f. Pearson correlation analyses between miR-138-5p expression and MIR17HG expression (e) or the HK1 IHC score (f) in 105 primary CRC tissues in the FUSCC dataset; g, h. Levels of miR-138-5p (g) and HK1 mRNA(h) in 8 adjacent normal, CRC and paired liver metastatic tissues; i. Immunohistochemical (IHC) score of HK1 in 8 adjacent normal, CRC and paired liver metastatic tissues; j. Representative HK1 immunohistochemical (IHC) images in adjacent normal, CRC and paired liver metastatic tissues; original magnification, 200 ×; scale bar, 100 μm; k, l. Expression and recurrence free survival analysis for miR-138-5p (k) and HK1 (l) in colorectal cancer panel using public datasets. *P < 0.05; **P < 0.01; ***P < 0.001; ns, not significant.

Supplementary Fig. S9. Lactate promotes MIR17HG transcription by activating the p38/Elk-1 pathway. a, b. A luciferase reporter vector (pNF-κB) containing an NF-κB response element was used to evaluate NF-κB activity after lactate stimulation (20 mM for 12 hours) in SW480 (a) and RKO (b) cells. PBS was used as the negative control, and TNF-α (10 ng/mL for 12 hours) was used as the positive control; c, d. A TOP/FOP luciferase reporter assay was used to evaluate Wnt pathway activity after lactate stimulation (20 mM for 12 hours) in

SW480 (c) and RKO (d) cells. PBS was used as the negative control, and LiCl (40 mM for 12 hours) was used as the positive control; e. Western blot analysis was used to evaluate the activation of p38, ERK1/2, and JNK upon lactate (20 mM) stimulation for 12 hours in SW480 and RKO cells; f, g. Effects of knocking down 8 candidate transcription factors on MIR17HG expression in SW480 (f) and RKO (g) cells stimulated with lactate. *P < 0.05; **P < 0.01; ***P < 0.001; ns, not significant.

Supplementary Fig. S10. Schematic of the mechanism by which MIR17HG functions as a ceRNA to promote colorectal cancer glycolysis and accumulated lactate upregulates MIR17HG expression via the p38/Elk-1 pathway. The red arrows with sharp ends indicate promotive effects, and the red arrow with flat heads indicate repressive effects.

Supplementary Table S1. Different expression of lncRNAs between primary tumor and adjacent normal tissues of 8 CRLM patients.

Supplementary Table S2. Different expression of lncRNAs between liver metastatic tumor and primary tumor tissues of 8 CRLM patients.

Supplementary Table S3. Different expression of lncRNAs between liver metastatic tumor and adjacent normal tissues of 8 CRLM patients.

Supplementary Table S4. Different expression of mRNAs between primary tumor and adjacent normal tissues of 8 CRLM patients.

Supplementary Table S5. Different expression of mRNAs between liver metastatic tumor and primary tumor tissues of 8 CRLM patients.

Supplementary Table S6. Different expression of mRNAs between liver metastatic tumor and adjacent normal tissues of 8 CRLM patients.

Supplementary Table S7. Comprehensive WGCNA identified gene modules involved in colorectal cancer liver metastasis.

Supplementary Table S8. Relationships between MIR17HG expression and clinical factors in 105 colorectal cancer patients.

Supplementary Table S9. Correlations between the clinicopathological characteristics and HK1 expression in 105 CRC patients.

Supplementary Table S10. Correlations between the clinic-pathological characteristics and miR-138-5p expression in 105 CRC patients.

Supplementary Table S11. Data of sequences for qPCR and cell transfection in this study.

Supplementary Table S12. Data of antibodies used in our research.

Supplementary materials and methods

Cell culture and vector construction

Eight CRC cell lines (Caco2, HT29, SW480, SW620, RKO, HCT116, HCT8, and DLD-1) were obtained from the Cell Bank of the Chinese Academy of Sciences, and one normal colon mucosal epithelial cell line (FHC) was obtained from ATCC. The cell lines were characterized using short tandem repeat markers and tested for mycoplasma contamination. Cells were cultured in DMEM (Gibco, Grand Island, NY, USA) containing 10% fetal bovine serum (FBS, Invitrogen, Camarillo, CA, USA) and 1% penicillin/streptomycin (Gibco, Grand Island, NY, USA). All cells were cultured at 37 °C in a humidified 5% CO_2 atmosphere. MiRNA mimics and inhibitors were purchased from RiboBio (Guangzhou, China). The lentiviral vectors used for overexpression or knockdown of MIR17HG and HK1 were synthesized by GenePharma (Shanghai, China). Stably transfected cell lines were selected using puromycin (Solarbio, Beijing, China) for 1 week.

Patient samples

For RNA sequencing, we collected adjacent normal tissues, CRC tissues, and paired liver metastatic tissues from 8 patients who underwent simultaneous resection of the CRC tumor and liver metastasis and did not receive any preoperative treatment. For survival analysis, a total of 105 patients pathologically diagnosed were enrolled in our study. All patients received treatment at Fudan University Shanghai Cancer Center (FUSCC) between 2008 and 2009. Patients who died from non-cancerous causes (such as car accidents and cardiovascular disease) were excluded. The entire patient cohort was divided into two groups according to the expression level of MIR17HG or miR-138-5p (high: fold change of equal to or greater

than the mean; low: fold change of less than the mean). For metabolic analysis, a total of 50 patients who underwent ^{18}F-FDG PET/CT examination at FUSCC between 2019 and 2020 were enrolled. Imaging diagnoses were processed independently by two radiologists. The sample size difference between each group was less than 3 times to ensure adequate power to detect a pre-specified effect. The present study was approved by the Ethics Committee of Fudan University Shanghai Cancer Center (FUSCC, ID: 050432-4-1911D), and informed consent was obtained from all patients before enrollment in this study.

Chromatin immunoprecipitation (ChIP) assay

We performed a ChIP assay to investigate the direct interaction between Elk-1 and the MIR17HG promoter using a Plus Enzymatic ChIP Kit (CST, Boston, MA, USA) according to the kit's reference guide. Briefly, we added 37% formaldehyde to a final concentration of 1% and incubated cells for 10 minutes at room temperature before glycine was added to terminate cross-linking. After mixing with the micrococcal nuclease provided in the kit, cells were incubated for 30 minutes at 37°C for DNA digestion, which was stopped by incubation with EDTA on ice for 2 minutes. For each immunoprecipitation reaction, an anti-Elk-1 antibody (2 μg) or a protocol-recommended internal control antibody was added to each microcentrifuge tube and incubated overnight at 4°C prior to incubation with the provided magnetic beads for 2 hours at 4°C with rotation. Then, elution of chromatin, reversal of cross-links, and DNA purification were sequentially performed as recommended. The enrichment degree was determined by quantitative real-time PCR. Each experiment was repeated five times.

Cell migration and invasion assay

To perform the wound healing assay, we marked the back of 6-well plates with

horizontal lines spaced 0.5~1 cm apart before seeding of cells in logarithmic phase, which made it easier to observe the same regions at different time points. We used a 200 μL pipette tip to scratch the cells in the wells, and the initial scratch area (0 h area) was assessed after washing 3 times with sterile PBS. Cells were incubated in a 5% CO_2 37°C atmosphere as usual, and we then measured and recorded the wound area after 24 hours. The migration ability was calculated with the following equation: migration ability = (0 h area - 24 h area)/0 h area. To measure the invasion ability of the indicated cells, 8.0 μm pore transwell inserts (Millipore, Billerica, MA, USA) were coated with Matrigel (BD Biosciences, Bedford, MA, USA), and CRC cells suspended in FBS-free medium were seeded into the upper chamber, while 20% FBS medium was added into the lower chamber. Cells were incubated for 24 hours, washed, fixed, stained with crystal violet, and observed under a light microscope. Each experiment was repeated five times. Data are presented as the mean ± SD values of the calculated migration ability.

Immunohistochemical (IHC) assay

Following dewaxing and hydration, sections were incubated with 3% hydrogen peroxide in a wet box for 10 minutes. After washing with PBS three times, antigen retrieval was performed in 10 mM citrate buffer (pH=6.0). Blocking of unspecific antigen binding sites using 10% normal goat serum was performed at room temperature for 1 hour. Subsequently, sections were incubated with a primary antibody purchased from Abcam (ab150423, 1:100) overnight at 4°C. Then, sections were incubated with an HRP-conjugated secondary antibody at room temperature for 1 hour and washed two times with PBS. Then, slides were stained with diaminobenzidine at room temperature for 10 minutes and counterstained with

hematoxylin.

Dual luciferase assay

To confirm the interaction between miR-138-5p and MIR17HG or HK1, wild-type or mutated fragments of the MIR17HG gene and the HK1 3'UTR with or without the putative miR-138-5p complementary sequences were synthesized by GenePharma (Shanghai, China) and inserted downstream of the firefly luciferase coding sequence in the pmirGLO vector. MiRNA mimics or negative controls (RiboBio, Guangzhou, China) were then cotransfected with recombinant pmirGLO plasmids into different cell clones with Lipofectamine 3000 (Invitrogen, Carlsbad, CA, USA). Detection of luciferase activity was performed 48 hours after transfection. Different wild-type and mutant fragments of the MIR17HG promoter were synthesized and cloned into the pGL4.20 vector, which was cotransfected with the pRL-TK plasmid into the indicated cells at a 5:1 ratio. To investigate whether lactate can activate the NF-κB or Wnt/β-catenin signaling pathway, a pNF-κB reporter plasmid (Beyotime Biotechnology, Shanghai, China) and a TOP-Flash/FOP-Flash vector (YouBio, Hunan, China) were used. The luciferase activities were determined by a dual luciferase reporter assay system (Promega, Madison, WI, USA). Renilla fluorescence was used as the internal control to calculate the relative firefly luciferase activity.

Biotinylated miRNA pulldown assay

SW480 and RKO cells were transfected with biotinylated miR-138-5p mimics or miR-138-5p mutants using Lipofectamine 3000 (Invitrogen, Carlsbad, CA, USA), collected 48 hours after transfection, washed with PBS, and lysed for 15 minutes on ice. After centrifugation, 100-μl aliquots of the lysates were used as input. All other samples were

incubated with M-280 streptavidin-coupled Dynabeads (Invitrogen, Carlsbad, CA, USA) at 4°C for approximately 3.5 hours and were then eluted with wash buffer. Then, TRIzol (Beyotime Biotechnology, Shanghai, China) was used to purify RNA for subsequent analysis. The biotinylated miR-138-5p mimics and mutants were synthesized by RiboBio (Guangzhou, China).

RNA immunoprecipitation (RIP) assay

MiR-138-5p mimics and the miR-138-5p mutant were transfected into cells using Lipofectamine 3000 (Invitrogen, Carlsbad, CA, USA). AGO2 immunoprecipitation was performed 48 hours after transfection using an RNA immunoprecipitation kit (GeneSeed, Guangzhou, China) according to the kit's instructions. IgG (ab172730) and anti-AGO2 (ab32381) antibodies were purchased from Abcam (Cambridge, MA, USA). Input was used as the positive control, and IgG was used as the negative control. Western blot analysis with the anti-AGO2 antibody confirmed the efficiency of immunoprecipitation, and the harvested RNAs were analyzed by quantitative reverse transcription PCR.

Glucose uptake, lactate production and ATP production assays

CRC cells were seeded into a 96-well plate and were then analyzed using a colorimetric glucose uptake assay kit (AAT Bioquest, Sunnyvale, CA, USA) and a colorimetric L-lactate assay kit (AAT Bioquest, Sunnyvale, CA, USA) according to the manufacturer's protocols. For the ATP assay, cells cultured in 6-well plates were lysed in 200 μL/well lysis buffer on ice and centrifuged at 4°C. The ATP content in the supernatant was determined using an enhanced ATP assay kit (Beyotime Biotechnology, Shanghai, China) in adherence to the recommended protocol. The content was normalized to the cell number. Each experiment was repeated five

times.

Extracellular acidification rate assay

The extracellular acidification rate (ECAR) was measured using a Seahorse XFe24 extracellular flux analyzer (Seahorse Bioscience, Billerica, MA, USA) according to the manufacturer's guidelines. Briefly, on the day before the ECAR assay, the sensors were submerged in the calibrant and placed in a non-CO_2 37°C incubator overnight for improved hydration. Cells were seeded into a 24-well plate, and medium was added to a final volume of 250 μL/well after 5 hours. After overnight incubation, assay medium containing 2 mM glutamine was used to rinse cells two times and was added to a final volume of 500 μL/well. Then, cells were incubated in a non-CO_2 37°C atmosphere for 1 hour prior to the assay. Glucose (10 mM), oligomycin (1 μM), and 2-DG (100 mM) were injected sequentially to measure the ECAR with 3 repeated tests after each injection. Each point represents the average of three independent samples.

In vivo experiment

Spleen injection, with the highest success rate is the most effective method for the construction of CRC liver metastasis causing little damage to mice [1]. To establish liver metastasis models, CRC cells (1×10^7 cells/200 μL) were injected into the spleens of female BALB/c-nude mice (six weeks of age, 15 mice/group). Then, five randomly selected mice from each group were sacrificed, and the livers were collected for hematoxylin and eosin (HE) staining at week 6. The liver metastatic colonies were counted under a light microscope. The rest of the mice in each group were observed until week 12 for survival analysis. Mice that had died from other causes (such as fighting and infection) were excluded. Our experiments

were approved by the Animal Experiment Ethics Committee of FUSCC.

Statistical analysis

Quantitative data for two or more than two groups were analyzed using two-sided t-test or one-way ANOVA, respectively. The variance was similar between the groups that were statistically compared using t-test. Qualitative data were analyzed by the chi-square test or Fisher's exact test. The Kaplan-Meier method with the log-rank test was used for survival analysis. Correlations were analyzed by Pearson's or Spearman's test. R program 3.0.1 was used for comprehensive WGCNA. SPSS v25 (IBM Inc., Armonk, NY, USA) was used for all analyses, and Prism v8 (GraphPad Software Inc., San Diego, CA, USA) was employed for plotting. All experiments were repeated at least three times, and the data are shown as the mean ± standard deviation (SD) values. $P<0.05$ was considered to indicate statistical significance.

References

1. Peng K, Kou L, Yu L, Bai C, Li M, Mo P, et al. Histone demethylase JMJD2D interacts With β-catenin to induce transcription and activate colorectal cancer cell proliferation and tumor growth in mice. Gastroenterology. 2019;156:1112-26.
