# Supplementary material for: LncRNA MIR17HG promotes colorectal cancer liver metastasis by mediating a glycolysis-associated positive feedback circuit
Source: Oncogene. 2021 Jun 18;40(28):4709–24. doi: 10.1038/s41388-021-01859-6 (PMC8282501; doi:10.1038/s41388-021-01859-6)
Supplement: Supplementary file 14 — Description of Supplementary Materials [file 41388_2021_1859_MOESM14_ESM.docx]

The PDF document ***Supplementary Information*** contains the following sections:

1. Supplementary Fig. S1-S10;
2. Legends of Supplementary Fig. S1-S10;
3. Headings of Supplementary Table S1-S12;
4. Supplementary materials and methods.

Supplementary Table S1-S12 are displayed in separate Excel files.
